# Large-scale proteomics analysis of five brain regions from Parkinson’s disease patients with a *GBA1* mutation

**DOI:** 10.1038/s41531-024-00645-x

**Published:** 2024-02-08

**Authors:** Shani Blumenreich, Tamar Nehushtan, Meital Kupervaser, Tali Shalit, Alexandra Gabashvili, Tammar Joseph, Ivan Milenkovic, John Hardy, Anthony H. Futerman

**Affiliations:** 1 Department of Biomolecular Sciences, Rehovot, 76100 Israel; 2 Nancy and Stephen Grand Israel National Center for Personalized Medicine, Weizmann Institute of Science, Rehovot, 76100 Israel; 3 Department of Neurology, Medical University of Vienna, Vienna, Austria; 4 Department of Neurogenerative Disease, UCL Dementia Research Institute, University College London, London, WC1N 3BG UK; 5 The Joseph Meyerhof Professor of Biochemistry at the Weizmann Institute of Science, Rehovot, Israel

**Keywords:** Biochemistry, Neurological disorders

## Abstract

Despite being the second most common neurodegenerative disorder, little is known about Parkinson’s disease (PD) pathogenesis. A number of genetic factors predispose towards PD, among them mutations in *GBA1*, which encodes the lysosomal enzyme acid-β-glucosidase. We now perform non-targeted, mass spectrometry based quantitative proteomics on five brain regions from PD patients with a *GBA1* mutation (PD-GBA) and compare to age- and sex-matched idiopathic PD patients (IPD) and controls. Two proteins were differentially-expressed in all five brain regions whereas significant differences were detected between the brain regions, with changes consistent with loss of dopaminergic signaling in the substantia nigra, and activation of a number of pathways in the cingulate gyrus, including ceramide synthesis. Mitochondrial oxidative phosphorylation was inactivated in PD samples in most brain regions and to a larger extent in PD-GBA. This study provides a comprehensive large-scale proteomics dataset for the study of PD-GBA.

## Introduction

Parkinson’s disease (PD) occurs in up to 3% of the aged population above the age of 80^1^, and is typically characterized by loss of dopaminergic neurons in the substantia nigra (SN) and accumulation of α-synuclein in a number of brain regions^2^. The ultimate cause of PD is unknown, but in the past couple of decades a number of genetic factors have been shown to predispose towards the development of PD, including mutations in *GBA1*, which encodes the lysosomal enzyme acid-β-glucosidase (GCase)^3^. Homozygous mutations in *GBA1* cause Gaucher disease, but individuals with a mutation in only one allele do not show any Gaucher disease symptoms^4^. However, both homo- and heterozygous mutations in *GBA1* increase the likelihood of developing PD by a significant extent. While this genetic association is well established, little is known about the mechanistic association between *GBA1* mutations and PD^5^, although recent studies are consistent with the notion that one or other aspect of lysosomal function may be defective when *GBA1* or other genes encoding lysosomal enzymes are mutated^6^, which also predispose to PD^7,8^.

A number of approaches have been used to attempt to delineate the mechanistic relationship between PD and *GBA1* mutations, among them, high throughput omics^9–12^. Recently we used a unique tissue collection of brain tissues from idiopathic PD (IPD) patients and PD patients carrying a *GBA1* mutation (PD-GBA) to perform a non-targeted lipidomics analysis of four different brain regions^13^, namely the striatum (STR), which is part of the nigrostriatal dopaminergic pathway, and also the occipital cortex (OCC), middle temporal gyrus (MTG) and cingulate gyrus (CG). No changes in levels of glucosylceramide (GlcCer), the substrate of GCase, were observed although levels of gangliosides (sialic acid-containing glycosphingolipids) were elevated in 3 of the 4 brain regions in such a way that suggests upregulation of the pathway of ganglioside metabolism rather than modulation of the activity of specific enzymes in the pathway.

In the current study, we use the same tissue collection^13^ but with the inclusion of the substantia nigra (SN), and perform non-targeted, mass spectrometry based quantitative discovery proteomics of all five brain areas. Although a number of studies of human PD brain have been performed (reviewed in^14,15^), very few used more than one brain region from the same patients to allow comparison of possible changes across the same brain. Moreover, most have focused on the SN or on the cortex, with others using cerebrospinal fluid or blood serum. Of most importance for the current study is that no systematic studies are available comparing proteomics data from IPD with PD-GBA patients. Analysis of the data obtained in our current study confirms some pathophysiological pathways previously identified in studies of PD brain tissue, but also suggests brain region-specific changes that might be of great relevance for developing a more holistic approach to brain pathophysiology in IPD and PD-GBA patients than normally pursued.

## Results

### Study design

**Fig. 1:**
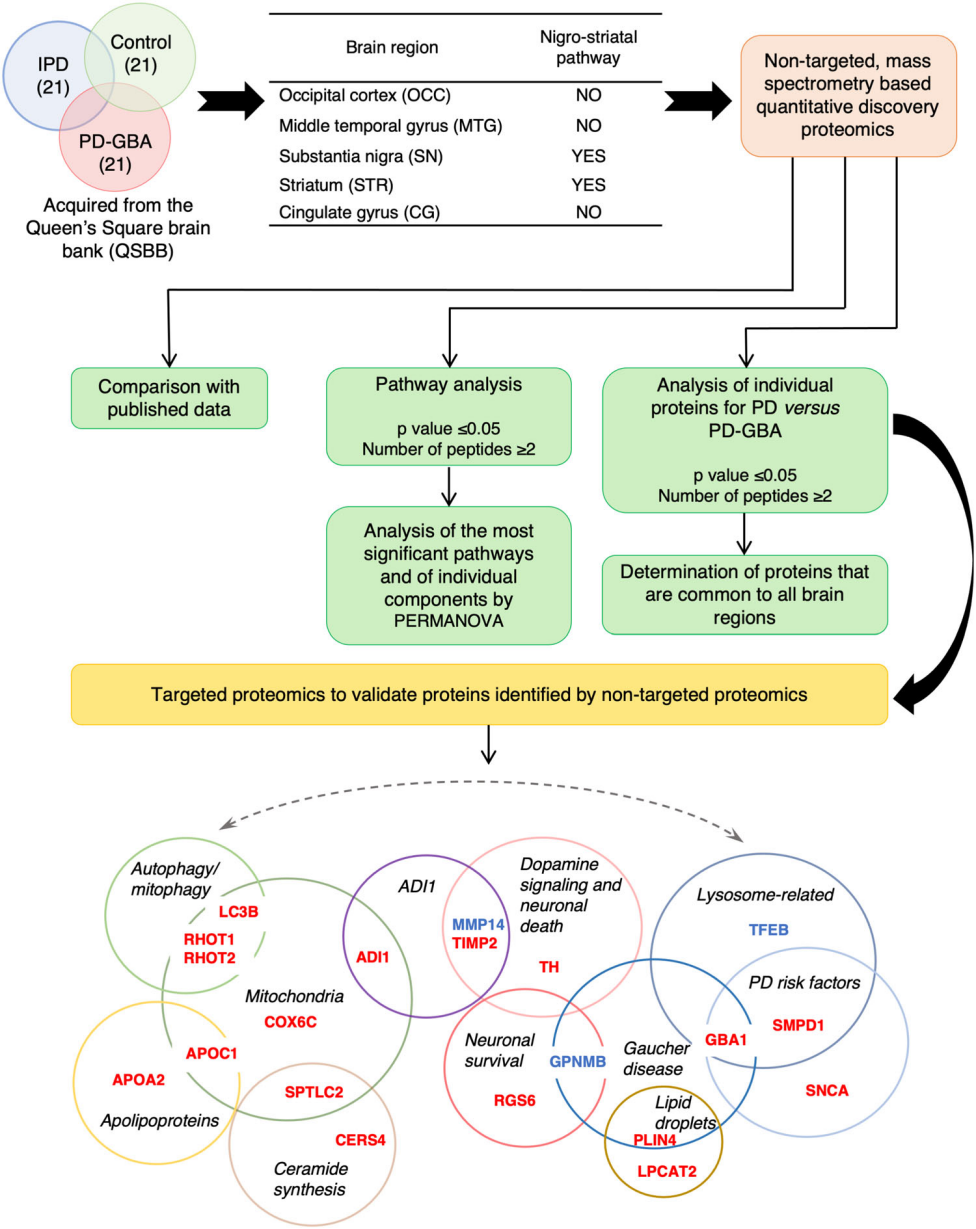
Study design. Non-targeted, mass spectrometry based quantitative discovery proteomics was performed on five human brain regions, namely the OCC, MTG, CG, STR and SN of 21 control, 21 IPD and 21 PD-GBA samples. Two of these regions are part of the nigrostriatal pathway and three are not. Subsequent to data collection, analysis was performed as indicated (*green* boxes). 20 proteins were chosen for targeted proteomics (*yellow*) in three brain regions (the CG, STR and SN), using the various criteria indicated in the text. Proteins in *red* were altered in at least one brain region in the non-targeted analysis; proteins in *blue* were selected as they directly impinge on the pathways discovered in the non-targeted study. The proteins are sorted according to the predominant pathway in which they are involved (*black*).

The goal of this study was to systematically examine pathophysiological pathways in IPD and PD-GBA brain by performing non-targeted, mass spectrometry-based quantitative discovery proteomics followed by targeted proteomics of selected proteins for validation (Fig. 1). This unique tissue sample set included 21 IPD patients without a *GBA1* mutation, 21 PD-GBA patients and 21 controls from five different brain regions (OCC, MTG, CG, STR and SN). The CG region contained only 52 samples (15 controls, 18 IPD and 19 PD-GBA), the STR region contained 61 samples (19 controls, 21 IPD and 21 PD-GBA) and the SN region contained 60 samples (20 controls, 20 IPD and 20 PD-GBA), for a total of 299 samples, a comparatively large number for human brain samples. Of these brain areas, 2 are associated with the nigrostriatal pathway (the SN and STR), while the other 3 regions all display some pathology in PD^16,17^ which may be responsible for non-motor symptoms (NMSs)^18^.

The groups were age- and sex-matched with no significant differences in the cause of death (control patients died from unrelated causes, whereas IPD and PD-GBA patients died from one or other issue related to PD). The PD patients were classified as IPD and most displayed characteristic Lewy body (LB) pathology. For PD-GBA brains^13^, *GBA1* mutations included, among others, patients with the N370S and the L444P mutations which are found at high levels in association with PD^3^. The same samples were used for a recent non-targeted lipidomics study, in which elevation of levels of gangliosides was detected in four brain regions^13^, although no significant changes were observed in levels of GlcCer.

Subsequent to the non-targeted, mass spectrometry-based quantitative discovery proteomics, and based on the data described below, 20 proteins were chosen for validation by targeted proteomics using parallel reaction monitoring (PRM) (Fig. 1), in which samples were spiked with heavy labeled synthetic peptides (three per protein) to facilitate their identification. The proteins were chosen based on either changes in their levels in the non-targeted analysis (17 of the proteins; see Supplementary Fig. 1 for a comparison of the two methods) or on their possible involvement in one or other of the putative pathological pathways (3 of the proteins); the latter included transcription factor EB (TFEB) [a master regulator of transcription of lysosomal proteins^19^], matrix metalloproteinase 14 (MMP14) [a protein that is directly regulated by one of the proteins detected in the non-targeted analysis, namely 1,2-dihydroxy-3-keto-5-methylthiopentene dioxygenase (ADI1^20^)] and transmembrane glycoprotein NMB (GPNMB) [a marker of the severity of symptoms in nGD^21^]. Targeted proteomics was conducted on three brain regions, the SN, STR and CG.

### Data quality and sample validation

**Fig. 2:**
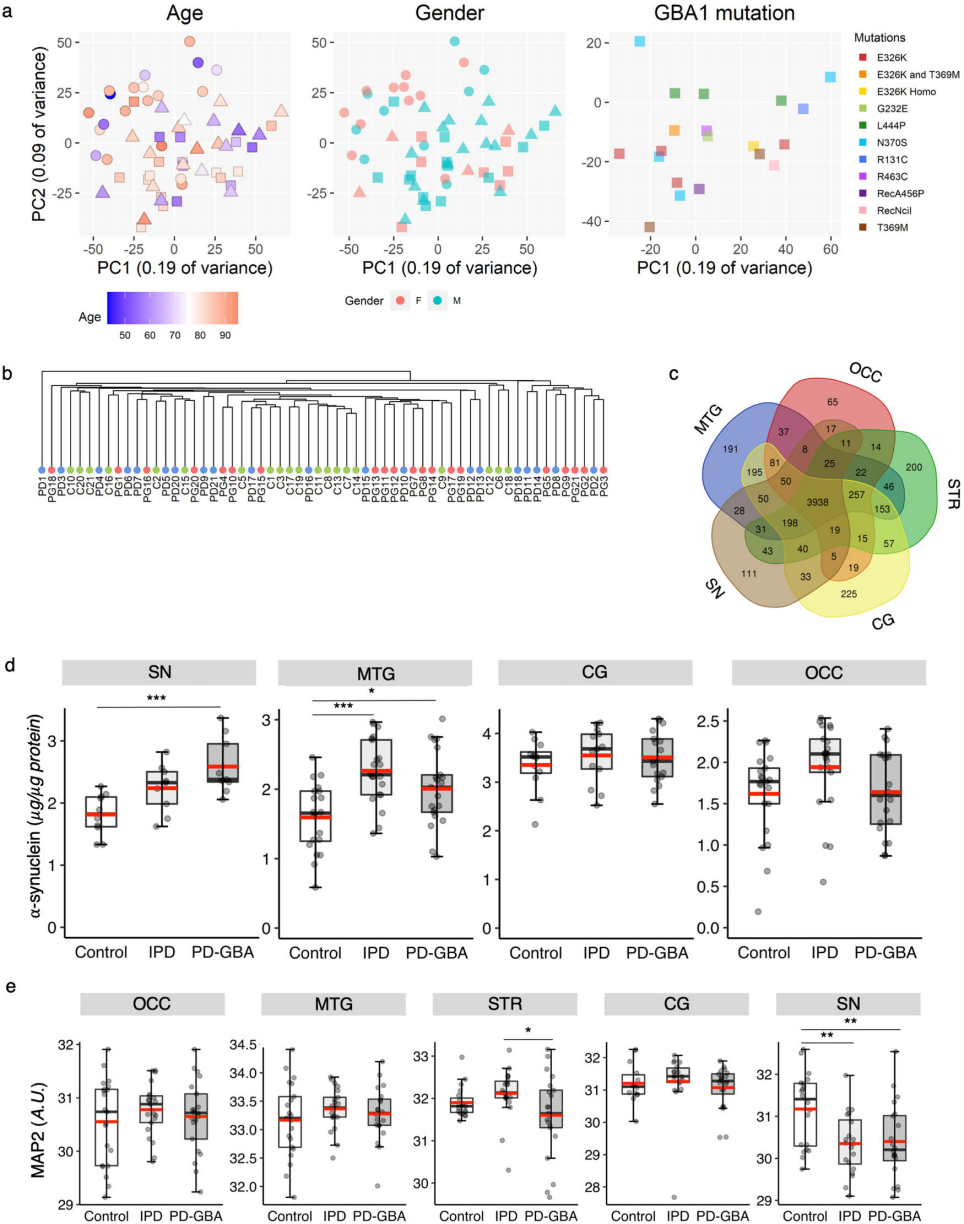
Evaluation of the quality of the non-targeted, mass spectrometry-based quantitative discovery proteomics. **a** PCA plots showing sample clustering according to gender, age and *GBA1* mutation for the SN; similar results were obtained for the other four brain regions (Supplementary Fig. 2). Controls, *circles*; IPD, *triangles*; PD-GBA, *squares*. The *y* and *x* axes represent sample variance. The color code is indicated for each panel (age, gender and *GBA1* mutation). No clustering into groups was observed for any of these factors. **b** Sample clustering for the SN; similar results were obtained for the other regions. No clear clustering into sample groups (i.e. control; C, IPD; PD or PD-GBA; PG) was observed. **c** Venn diagram showing the number of detected proteins in each brain region and the overlap between different regions. Thus, 3938 proteins were detected in total in the five brain regions and, for instance, 111 proteins were found exclusively in the SN and 191 in the MTG. **d** Levels of β-sheet oligomers of human α-synuclein measured by ELISA using monoclonal antibody 5G4 (which specifically identifies aggregated α-synuclein)^42,49^ from all available samples from the OCC, MTG and CG and *n* = 12 for SN in each group. **e** Levels of MAP2 in five brain regions from the non-targeted proteomics. Boxes represent lower quartile, median and upper quartile (*black*). The whiskers represent the minimum and maximum values, up to 1.5-times the interquartile range from the bottom or the top of the box to the furthest data point within that distance, thus excluding outliers. The mean is in *red*. For **d**, the *y* axis is in μg/μg protein. For **e**, the *y* axis is in arbitrary units. *p ≤ 0.05; **p ≤ 0.01; ***p ≤ 0.001, calculated using either ANOVA, followed by post-hoc pairwise comparisons, for the ELISA analysis or empirical Bayes moderation for the proteomics analysis.

Prior to detailed data analysis, quality control was performed on all of the samples. First, principal component analysis (PCA) on all or most proteins detected in each region (OCC, 4411 proteins; MTG, 5002 proteins; STR, 4714 proteins; CG, 5082 proteins; SN, 4534 proteins), revealed that samples did not cluster according to age, gender or *GBA1* mutation (in the case of PD-GBA samples) (Fig. 2a and Supplementary Figs 2 and 3), demonstrating that none of these factors influence the data. Each sample was also analyzed for the number of missing values (Supplementary Fig. 4), and in rare cases when a sample displayed a large number of missing values, it was removed from the analysis (where a missing value could either be due to a technical error or was close to or below the limit of detection), such as in the case of sample PG11 from the MTG and samples C13 and C15 from the CG. No clustering between the different samples was detected (Fig. 2b, Supplementary Fig. 5) which is not surprising since 42 of the 63 samples come from PD patients (with and without *GBA1* mutations); the fact that control samples did not cluster can likely be explained by the high variability in human brain samples between individual patients and the relatively small number of changes in protein levels (see below). Next, the number of overlapping proteins between each brain region was analyzed (Fig. 2c), with most of the identified proteins detected in all regions, permitting their evaluation and comparison in different brain regions.

An important factor in PD is the spread of α-synuclein, which is used to determine disease severity according to Braak staging^16^. Levels of β-sheet oligomers of α-synuclein, an aggregated form of α-synuclein, were analyzed by ELISA in all available control, IPD and PD-GBA samples for three brain regions and in 12 samples for the SN. According to Braak staging, the deposition of aggregated α-synuclein is observed in the SN in stage 3, in the MTG in stage 4, and in the OCC and CG in stage 5/6^16^. In the PD samples used for the proteomics analysis, β-sheet oligomers of α-synuclein accumulated in the SN and in the MTG, but not in the CG and OCC (Fig. 2d), suggesting that the samples were mainly taken from stage 4 patients. Levels of oligomers were higher in the SN of PD-GBA patients than in IPD and lower in the MTG than in IPD (Fig. 2d).

We also analyzed levels of MAP2, a protein found in the dendrites of neurons^22^. MAP2 levels decreased significantly in the SN, where neuronal loss is extensive in PD^2^, in both the IPD and PD-GBA groups (Fig. 2e), and to a somewhat greater extent in the STR in PD-GBA compared to IPD. No reduction in MAP2 levels were observed in the other 3 brain regions indicating little or no neuronal loss in these regions.

**Fig. 3:**
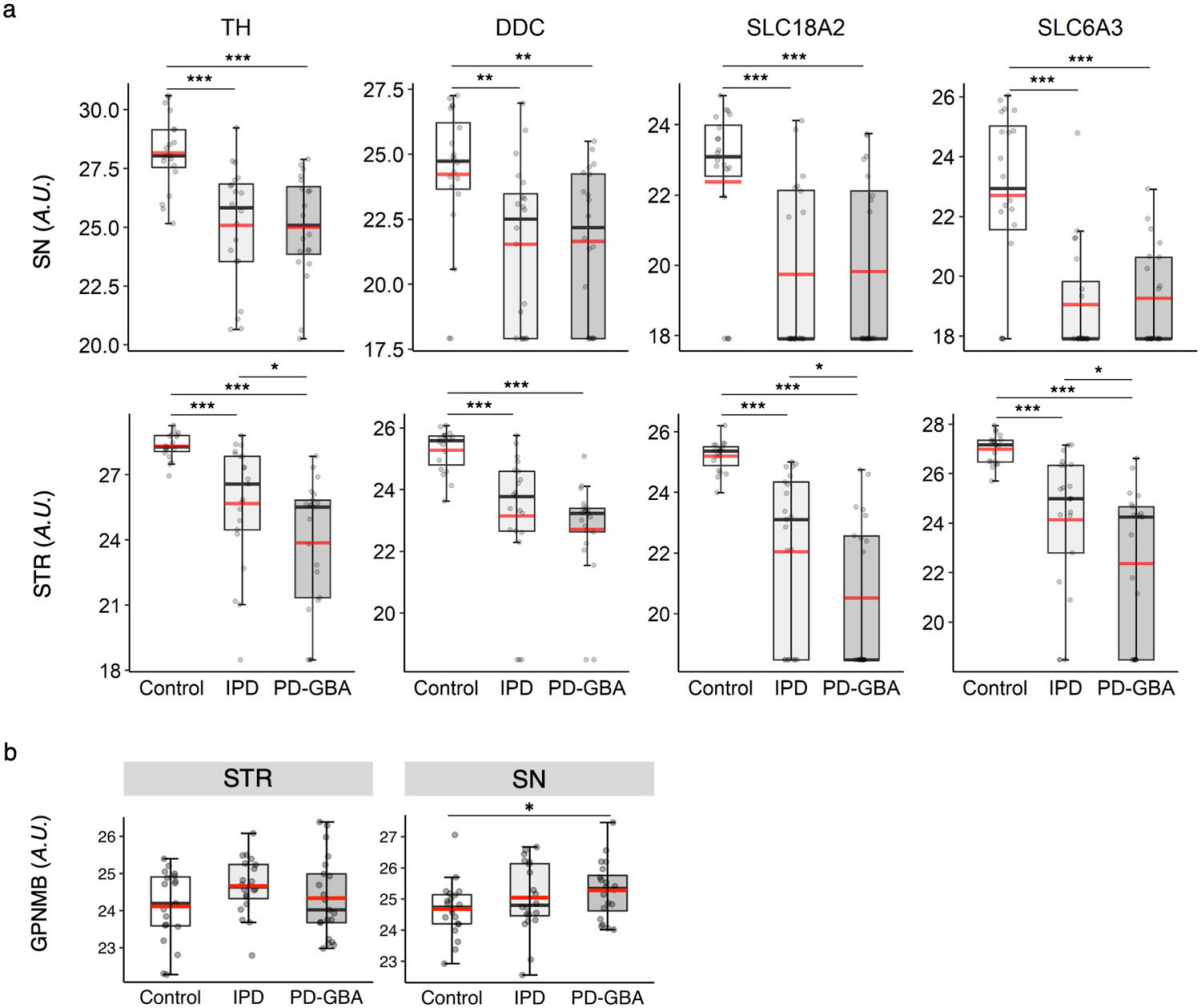
Analysis of proteins associated with dopaminergic pathways shows a more significant loss of these components in PD-GBA compared to IPD in the STR. **a** Boxplots of levels of four key proteins related to the dopaminergic pathway in the SN (*upper panel*) and STR (*lower panel*) from the non-targeted proteomics. **b** GPNMB levels detected by targeted proteomics in the STR and SN. Boxes represent lower quartile, median and upper quartile (*black*). The whiskers represent the minimum and maximum values, up to 1.5-times the interquartile range from the bottom or the top of the box to the furthest data point within that distance, thus excluding outliers. The mean is in *red*. The *y* axis is in arbitrary units. *p ≤ 0.05; **p ≤ 0.01; ***p ≤ 0.001, calculated using empirical Bayes moderation.

Since neuronal loss occurs concomitantly with the loss of components of the dopaminergic pathway in PD, we examined levels of 4 proteins involved in this pathway, tyrosine hydroxylase (TH), dopa decarboxylase (DDC), solute carrier family 18 member A2 (SLC18A2) and solute carrier family 6 member 3 (SLC6A3) (Fig. 3a). A significant reduction in levels of all 4 proteins was observed in both the SN and the STR (the nigrostriatal regions which are most affected in PD^2^); note that these 4 proteins were not detected in the other 3 brain regions. The extent of reduction of these proteins was larger in the STR of PD-GBA samples compared to IPD (Fig. 3a), consistent with greater axonal damage in PD-GBA compared to IPD, and also consistent with suggestions that PD-GBA is a more severe form of IPD^23,24^. Levels of one of the proteins, namely TH, was analyzed by targeted proteomics and compared to the non-targeted data, which gave a good correlation in the STR and in the SN (Supplementary Fig. 6).

Next, levels of GPNMB, which can be used as a marker for determining the severity of nGD disease^21^, were increased in the SN in PD-GBA (GPNMB was analyzed by targeted proteomics since this protein was not detected in the non-targeted analysis) (Fig. 3b). In conclusion, proteomics analysis is consistent with the classification of the patient samples in the clinical data, and consistent with the notion that PD-GBA is a more severe form of PD than IPD.

### Brain region-specific similarities and changes in protein levels between IPD and PD-GBA

**Fig. 4:**
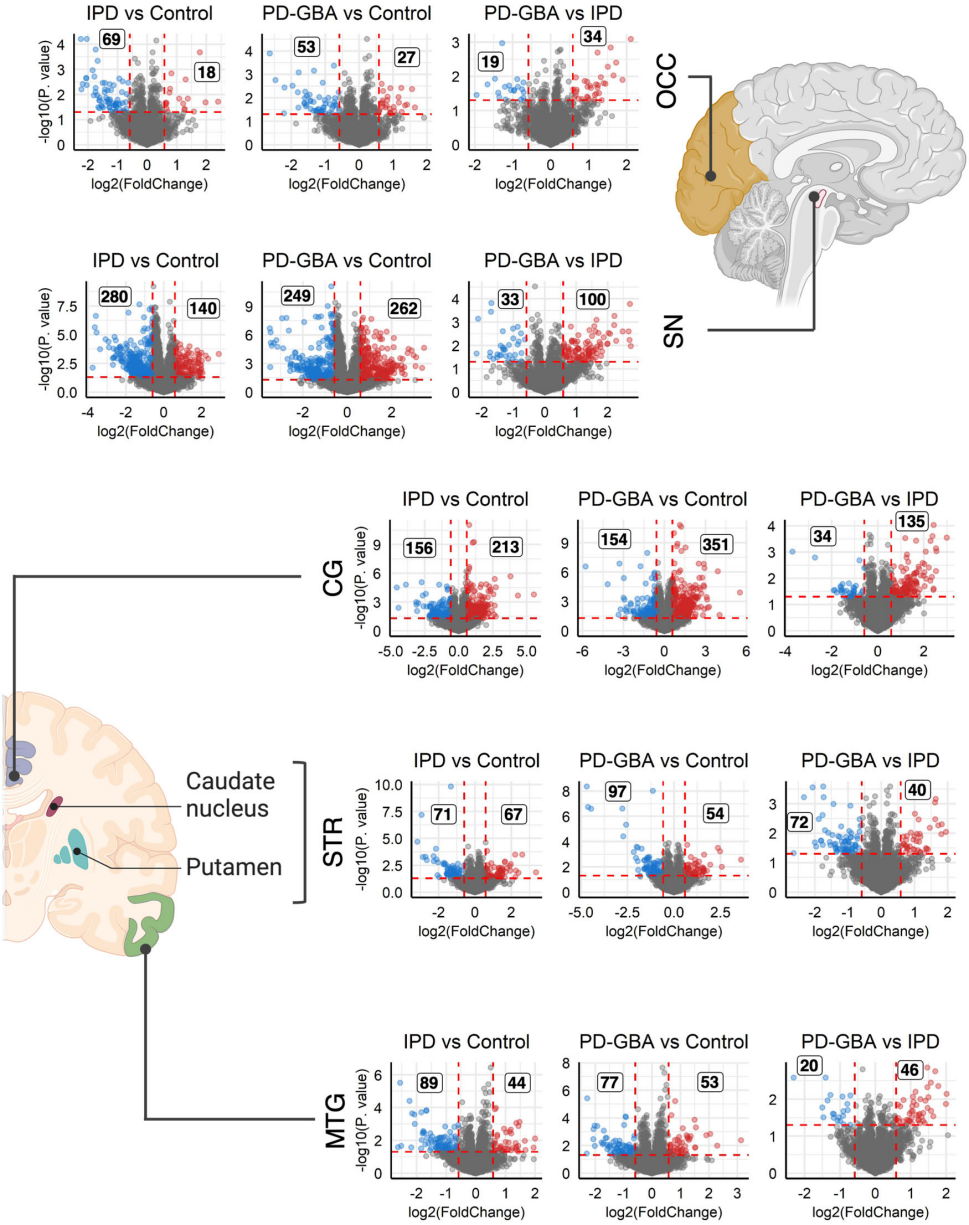
Differentially-expressed proteins in all five brain regions. Volcano plots using non-targeted proteomics data. The brain region is indicated (created using BioRender.com). Each plot shows a comparison between the three groups (i.e. IPD *versus* control, PD-GBA *versus* control or PD-GBA *versus* IPD). The dashed vertical lines (*red*) indicate fold-change ≥ 1.5 and the horizontal *red* lines indicate *p* ≤ 0.05, calculated using empirical Bayes moderation. Individual proteins are shown, with *red* indicating proteins whose levels were elevated and *blue* proteins whose levels were reduced, with the number of such proteins also indicated.

PD is characterized by loss of neurons in the SN. The SN projects to the STR and a significant loss of these projected axons is observed in the STR, which causes a lack of dopamine in this region, leading to pathology. However, other brain regions are affected in PD pathogenesis, such as the three other tissues chosen for this study, the OCC, MTG and CG. These brain regions exhibit altered functional connectivity^17^, display Lewy body pathology at different disease stages^16^ and are responsible for some of the NMSs observed in PD^25^. Analysis of changes in protein levels in all five regions indicate that the SN and CG display the largest number of changes in protein levels (Fig. 4). As might be expected based on PD pathology, the highest number of changes in protein levels is in the SN, with more proteins downregulated in IPD *versus* control (280 downregulated and 140 upregulated) but a similar number is found for up- or downregulated proteins in PD-GBA *versus* control (249 downregulated and 262 upregulated). Perhaps more unexpectedly, the next most affected region was the CG, in which differentially-expressed proteins are more upregulated in both PD groups (IPD *versus* control, 156 downregulated proteins and 213 upregulated; PD-GBA *versus* control, 154 downregulated and 351 upregulated). The relatively large extent of changes in the CG is consistent with the overactivation of this brain region in PD, as shown using functional magnetic resonance imaging^26^. The STR is not one of the most altered regions, though it is one of the most affected regions in PD. In all regions except for the STR, more proteins were upregulated then downregulated in PD-GBA *versus* IPD.

**Fig. 5:**
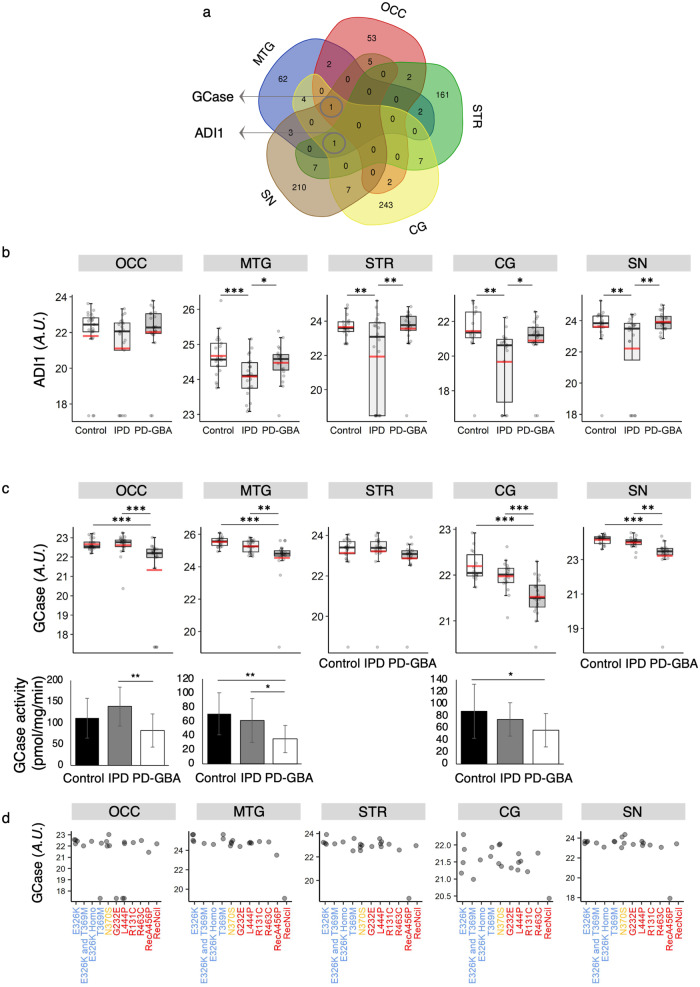
Two proteins are differentially expressed in all five brain regions. **a** Venn diagram of differentially expressed proteins between IPD and PD-GBA. Proteins which were differentially expressed between IPD and PD-GBA and between one of the PD groups *versus* the control in all five brain regions are indicated. Number of peptides ≥ 2, *p* ≤ 0.05. **b** Boxplots displaying non-targeted proteomics results of ADI1 levels in all five brain regions. **c** Boxplots (upper panel) displaying nontargeted proteomics of GCase levels in all brain regions measured in the analysis. For both **b** and **c**, the box represents lower quartile, median and upper quartile (*black*). The whiskers represent the minimum and maximum values, up to 1.5 times the interquartile range from the bottom or the top of the box to the furthest data point within that distance, thus excluding outliers. The mean is shown in *red*. For **b** and **c** (upper panel), the y axis is in arbitrary units. *p ≤ 0.05; **p ≤ 0.01; ***p ≤ 0.001, calculated using empirical Bayes moderation. GCase activity (*lower panel*) (*n* = 12 for control, IPD and PD-GBA, chosen blindly); *p ≤ 0.05; **p ≤ 0.01; ***p ≤ 0.001, calculated using ANOVA, followed by post-hoc pairwise comparisons. For **c** (lower panel), the y axis is in pmol/mg/min. Error bars indicate +/- standard deviation. **d**, The non-targeted proteomics data from **c** is plotted according to *GBA1* mutation classified as severe [G232E, R131C, L444P, R463C, RecA456P, RecNciI (*red*)], mild [N370S (*orange*)] and PD risk factors [E326K, T369M (*blue*)]^27,50,51^. Each point indicates GCase levels in an individual patient (arbitrary units).

Levels of only two proteins were altered in all five brain regions in one or other of the groups (Fig. 5a), namely GCase and ADI1, which were differentially expressed between IPD and PD-GBA in all brain regions (with statistical significance in four regions) (Fig. 5b, c). ADI1 has not been previously reported to play a role in PD pathogenesis, but its levels were reduced in IPD, although unexpectedly, not in PD-GBA. This data could not be validated by targeted proteomics since only one out of three peptides was detected, and this peptide was not one of the 8 detected peptides identified in the non-targeted analysis.

In contrast, GCase levels decreased in PD-GBA in all brain regions irrespective of the *GBA1* mutation (Fig. 5c). This is somewhat unexpected since most of the mutations in the PD-GBA group are point mutations which are not necessarily expected to affect protein expression or stability, particularly mutations which are risk factors for PD but are not known to cause GD, such as E326K or T369M^27^. The non-targeted analysis was validated by the targeted analysis (Supplementary Fig. 7). The reduction in GCase levels was also confirmed by GCase enzymatic assays inasmuch as PD-GBA samples had lower levels of GCase activity than either controls or IPD samples (*n* = 12 for each group; Fig. 5c). There was no observed association between protein levels detected by non-targeted proteomics and the *GBA1* mutation (Fig. 5d), and the same mutation sometimes gave different levels of GCase expression (see for instance GCase levels for the E326K mutation in the CG). Finally, a recent suggestion implied that GCase activity decreases with age^28^, but we did not detect an age-dependent decrease in GCase levels from control patients by non-targeted proteomics (Supplementary Fig. 8).

### Changes in cellular and metabolic pathways in IPD and PD-GBA

**Fig. 6:**
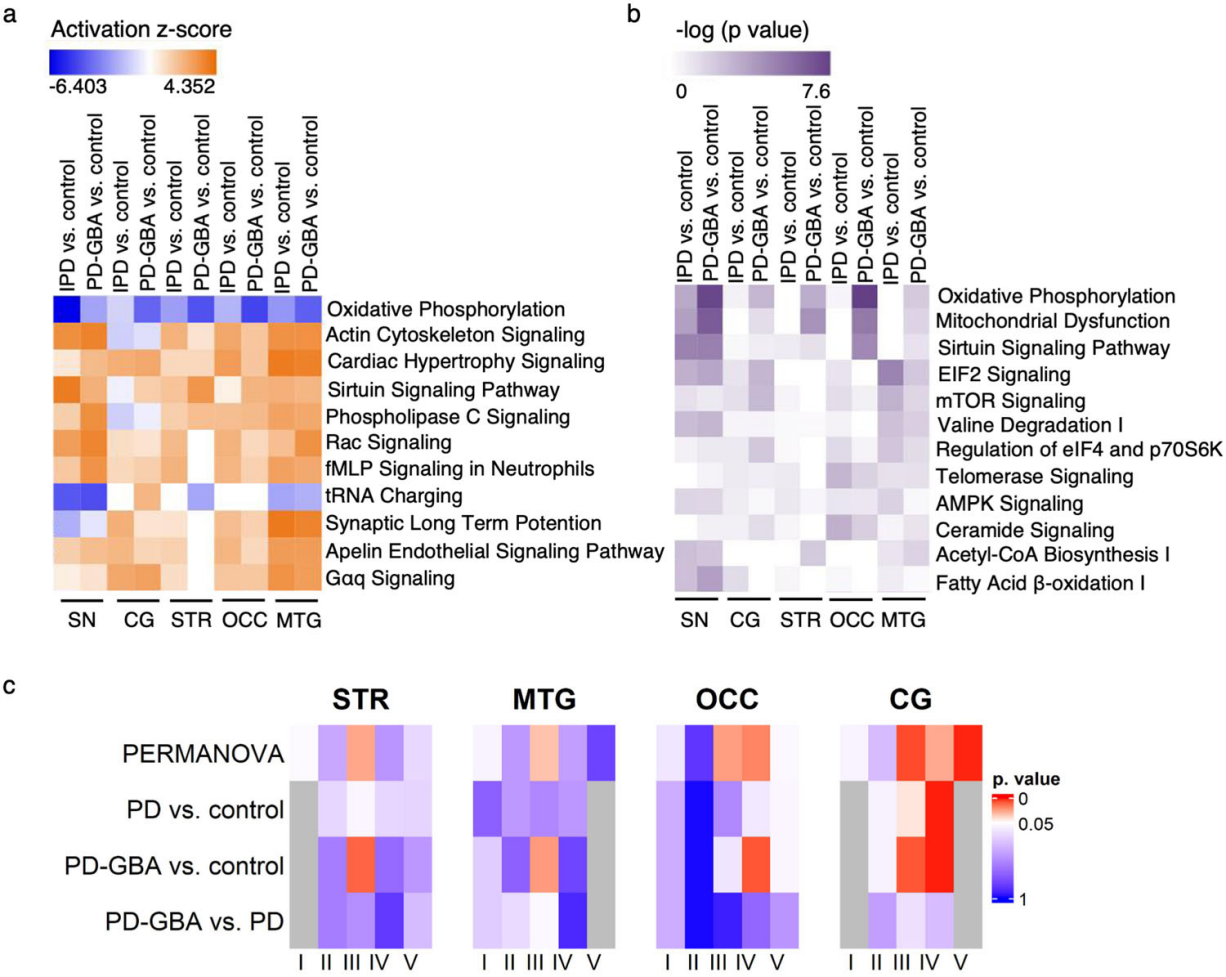
Mitochondrial pathways and specific respiratory electron transport complexes are reduced to a larger extent in PD-GBA than in IPD. Pathway analysis of the results of the five brain regions compared to each other, according to fold-change (**a**) and to *p* value (**b**). This analysis was conducted using IPA software, which also considers known inhibitors or activators of the pathway and scores the pathway according to the level of change in these factors, resulting in a predicted activation score of the pathway; a lower score means the pathway is inhibited and a higher score means the pathway is activated. IPA calculates a *p* value using the right-tailed Fisher’s Exact Test. For **a***, blue* indicates reduced activation of the pathway and *orange* indicates elevated activation of the pathway. For **b**, a darker shade of *purple* indicates higher statistical significance. Number of peptides ≥ 2, *p* ≤ 0.05. **c**, Analysis by PERMANOVA of mitochondrial complexes. This statistical test takes into account changes in all the subunits of each complex. *p* values are colored according to the scale shown, where values below 0.05 are in *red* and values above 0.05 are in *blue*. *Grey* indicates cases in which the number of subunits is greater than the number of tested samples (due to limitations of the PERMANOVA test). The SN was removed from the analysis as it included changes in all complexes in both groups, which is probably due to neuronal death in this region.

We next analyzed changes in cellular and metabolic pathways using ingenuity pathway analysis (IPA) (https://www.qiagenbioinformatics.com/products/ingenuitypathway-analysis). We first examined changes in each brain region independently, followed by a compared comparison of the altered pathways in all 5 regions, and expressed data as either fold-change (Fig. 6a) or *p* value, calculated using the right-tailed Fisher’s Exact Test (Fig. 6b). These two factors measure different parameters with fold-change (activation z-score) taking into account levels of activators and inhibitors of classified pathways and accordingly predict whether a pathway is activated or inactivated, whereas *p* value indicates how many differentially expressed proteins were altered; changes in both are highly suggestive that the pathway in question has been moderated under the specific experimental conditions.

Only two pathways were altered according to both parameters, namely oxidative phosphorylation (Fig. 6a, b), and sirtuin signaling, although changes in the latter were relatively small and not consistent. A number of pathways only changed in one of the analyses, including mitochondrial dysfunction and ceramide signaling (*p* value) along with tRNA charging (activation z-score). In the case of oxidative phosphorylation, the activation z-score, which takes into account expression levels of known inhibitors and activators of the pathway, suggests that the pathway was inactivated to a larger extent in IPD compared to controls and to an even larger extent in PD-GBA, with the exception of the SN where the pathway was affected more in IPD than in PD-GBA. Likewise, changes in oxidative phosphorylation were larger according to *p* value for PD-GBA than for IPD in all regions. Further analysis of the pathway of oxidative phosphorylation by PERMANOVA revealed that specific complexes were affected to a larger extent in PD-GBA than in IPD (specifically complexes III and IV) (Fig. 6c). Finally, targeted proteomics validated the non-targeted analysis for cytochrome C oxidase subunit 6 C (COX6C), a subunit of mitochondrial complex IV (Supplementary Fig. 9).

**Fig. 7:**
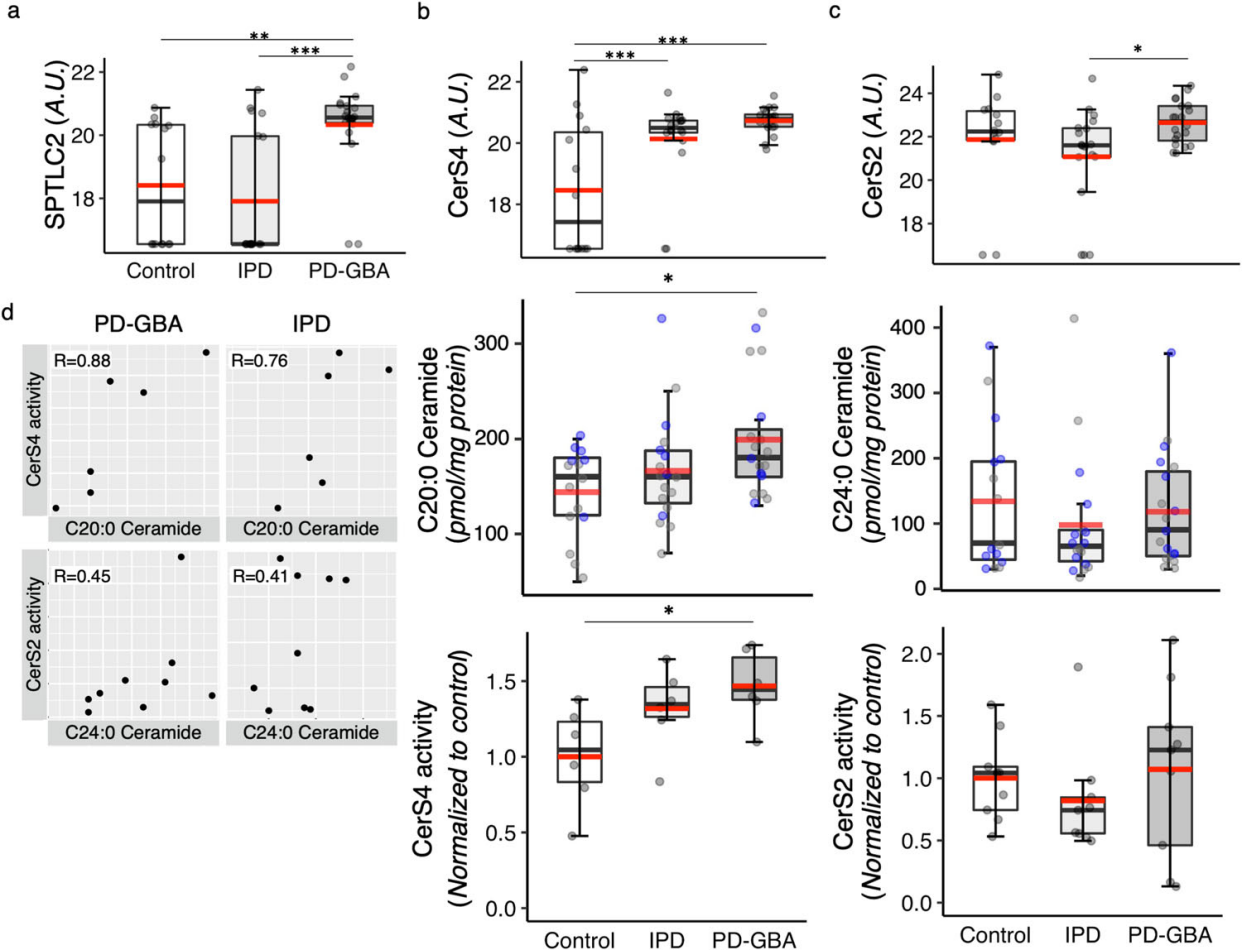
Elevation of the ceramide synthesis pathway in the CG of PD-GBA. **a** Boxplots indicating levels of SPTLC2 by non-targeted proteomics. **b** Boxplot of non-targeted proteomics for CerS4 levels, along with levels of C20-ceramide (data from ref. ^13^); purple indicates which samples were used for the CerS activity assay in the plot below (*n* = 6 for each group). **c** Boxplot of non-targeted proteomics of CerS2 levels in the CG with levels of C20:0-ceramide (taken from ref. ^13^) in the same samples, with purple indicating which samples were used for the CerS activity assay (*n* = 9 for each group). **d** Pearson correlation matrices of CerS4 activity and CerS2 activity compared with lipidomics data for PD-GBA and for IPD. Correlation coefficients are indicated. Axes represent CerS activity data (*pmol/mg protein/min*) *versus* the lipidomics data (*pmol/mg protein*). For all boxplots, the box represents lower quartile, median and upper quartile (*black*). The whiskers represent the minimum and maximum values, up to 1.5 times the interquartile range from the bottom or the top of the box to the furthest data point within that distance, thus excluding outliers. The mean is shown in *red*. *p ≤ 0.05, **p ≤ 0.01, ***p ≤ 0.001, calculated using either empirical Bayes moderation for the proteomics analysis or ANOVA, followed by post-hoc pairwise comparisons, for the activity assays.

One of the pathways, which was significantly affected was ceramide synthesis, at least in the CG (Table 1, Supplementary Table 1). Two subunits of serine palmitoyl transferase were down-regulated in IPD but either found at control levels (SPTLC1) or elevated in PD-GBA versus IPD (SPTLC1 and SPTLC2) (Table 1 and Fig. 7a); changes in SPTLC2 were validated by targeted proteomics (Supplementary Fig. 10A). Levels of three members of the ceramide synthase (CerS) family were elevated in either or both IPD or PD-GBA (CerS6, Table 1; CerS4 and CerS2, Table 1 and Fig. 7b, c), with CerS4 validated by targeted proteomics (Supplementary Fig. 10B) (CerS2 was not analyzed by targeted proteomics). Further validation was obtained by comparing C20- (generated by CerS4^29^) and C24-ceramide (generated by CerS2^30^) levels in the same samples (data taken from ref. ^13^) which, at least in the case of CerS4, revealed a significant elevation of C20-ceramide levels in PD-GBA (Fig. 7b). Finally, CerS4 and CerS2 activity measurements were consistent with the proteomics and lipidomics data (Fig. 7b, c) and there was a reasonable correlation between levels of ceramides in individual patients and CerS activity (Fig. 7d), Together, this data indicates that the ceramide synthesis pathway is altered in the CG of PD-GBA samples.

**Table 1:** Elevation of ceramide synthesis-related proteins in the CG of PD-GBA samples.

Gene	Protein	Ratio (IPD vs control)	Ratio (PD-GBA vs control)	Ratio (PD-GBA vs IPD)
*SPTLC1*	Serine palmitoyltransferase, long chain base subunit 1	0.38*	0.94	2.47*
*SPTLC2*	Serine palmitoyltransferase, long chain base subunit 2	0.71	3.79**	5.37***
*KDSR*	3-ketodihydrosphingosine reductase	1.11	1.50***	1.35**
*CERS1*	Ceramide synthase 1	0.93	1.02	1.10
*CERS2*	Ceramide synthase 2	0.58	1.72	2.97*
*CERS4*	Ceramide synthase 4	3.19***	4.82***	1.51
*CERS6*	Ceramide synthase 6	1.66	2.48**	1.49
*DEGS1*	Sphingolipid delta(4)-desaturase	1.04	1.14	1.09

Differentially expressed ceramide synthesis-related proteins in the CG. Ratios of protein levels from the non-targeted proteomics results are shown for IPD *versus* control, PD-GBA *versus* control and PD-GBA *versus* IPD. *p ≤ 0.05; **p ≤ 0.01; ***p ≤ 0.001, calculated using empirical Bayes moderation.

### Cellular changes are not restricted to any one organelle and do not differ between IPD and PD-GBA

**Fig. 8:**
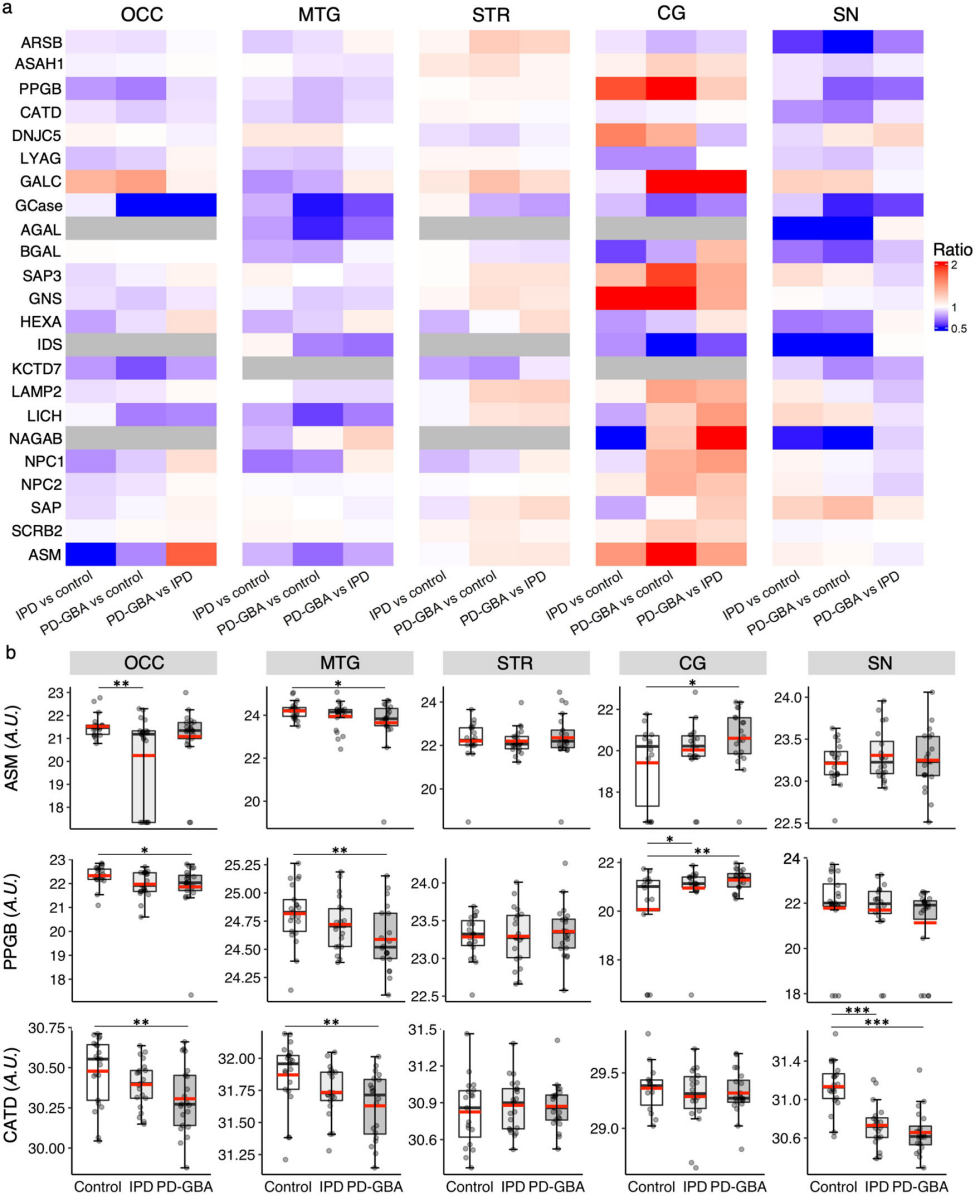
Alterations in levels of LSD-related proteins in IPD and PD-GBA. **a** Heatmap of LSD-related proteins which differ in at least one of the brain regions in any of the groups in the non-targeted proteomics. All five brain regions are displayed. Ratios of protein levels are shown for IPD *versus* control, PD-GBA *versus* control and PD-GBA *versus* IPD. *Blue* indicates a ratio of < 1 and *red* a ratio of > 1; *grey* indicates not detected. Reduced levels of these proteins are observed in most brain regions in both PD groups, with the exception of the CG, where they appear to be more elevated, especially in the PD-GBA group. **b** Boxplots displaying non-targeted proteomics data of ASM, PPGB and CATD levels in all five brain regions. The box represents lower quartile, median and upper quartile (*black*). The whiskers represent the minimum and maximum values, up to 1.5 times the interquartile range from the bottom or the top of the box to the furthest data point within that distance, thus excluding outliers. The mean is shown in *red*. The y axis is in arbitrary units. *p ≤ 0.05; **p ≤ 0.01; ***p ≤ 0.001, calculated using empirical Bayes moderation.

We next determined whether changes in levels of proteins associated with different subcellular locations differ between IPD and PD-GBA. Indeed, a recent study suggested an excessive burden of lysosomal storage disorder gene variants in PD^6^. Of the 54 proteins identified in this study^6^, levels of 23 were altered in one or other brain region in either IPD or PD-GBA by non-targeted proteomics (Supplementary Table 2), with most decreased, although many were elevated in PD-GBA in the CG (Fig. 8a). Levels of acid sphingomyelinase (ASM), which is also a risk factor for PD^31^, decreased in the IPD group in the OCC and in the PD-GBA group in the MTG, but was elevated in the CG in the PD-GBA group (confirmed by targeted proteomics; Supplementary Fig. 11). Similarly, levels of two other proteins, cathepsin A (PPGB) and cathepsin D (CATD) were reduced in the OCC, MTG and SN, whereas PPGB was elevated in the CG in both PD groups. While this data is not definitive about whether there are global changes in lysosomal proteins in IPD and PD-GBA, they are nevertheless consistent with the notion that the lysosome plays a critical role in PD pathology. However, upon differentiating between proteins, which are located in different intracellular compartments, the mitochondria, endoplasmic reticulum and plasma membrane all displayed a similar number of differentially expressed proteins as the lysosome (Table 2 and Supplementary Table 3).

**Table 2:** Several cell compartments are altered in both PD groups, but to a larger extent in PD-GBA in most brain regions.

	Mitochondria		Endoplasmic reticulum		Lysosome		Plasma membrane	
Brain region	Differentially expressed (IPD *versus* control)	Differentially expressed (PD-GBA *versus* control)	Differentially expressed (IPD *versus* control)	Differentially expressed (PD-GBA *versus* control)	Differentially expressed (IPD *versus* control)	Differentially expressed (PD-GBA *versus* control)	Differentially expressed (IPD *versus* control)	Differentially expressed (PD-GBA *versus* control)
OCC	9.0	8.5	6.5	5.5	3.8	6.6	6.4	5.9
MTG	14.6	14.8	12.3	13.6	6.0	14	9.8	13.9
STR	9.2	16.1	5.3	7.7	6.2	8.4	8.6	8.4
CG	15.4	20.2	21.2	27.8	18.9	26.9	17.2	22.4
SN	51.1	52.8	36.6	44.0	32.1	28.8	28.4	29.2

Percent of differentially expressed proteins out of the total number of the detected proteins related to four cell compartments (see Table S3); mitochondria, endoplasmic reticulum, lysosome and plasma membrane, in all five brain regions. Protein lists for each cell compartment were taken from “The human protein atlas” (https://www.proteinatlas.org/). *p* ≤ 0.05, calculated using empirical Bayes moderation, used as the cut-off for differential expression.

## Discussion

While the genetic connection between mutations in *GBA1*^3^, or in other genes encoding lysosomal proteins^6,8^ and PD is unambiguous, the mechanistic link is far less well understood. Many studies have approached this question by targeting components of the *GBA1*/lysosomal pathway^32^ with a view to delineating mechanistic pathways or to alleviating disease symptoms, but our approach has been to attempt to gain as wide a picture as possible by unbiased approaches such as lipidomics and proteomics analyses of brain tissue from IPD and PD-GBA patients. Thus, we collated a unique set of samples, from 5 different brain areas, in which some areas are directly associated with the nigrostriatal dopaminergic pathway, and some are not. Recently we completed a non-targeted lipidomics study^13^ and demonstrated no change in levels of GlcCer (the substrate of GCase), but significant changes in levels of gangliosides in most brain regions were observed. With this in mind, we further extended our analyses of this tissue collection by performing non-targeted proteomics analyses, as reported herein. The first stage in such a large-scale analysis is validation of data and sample quality. Indeed, analysis of dopaminergic components, and of α-synuclein levels support the designation of the samples, inasmuch as these components were altered as might be expected in PD samples, and likewise, analysis of GCase activity and levels were consistent with the genetic designation of the PD-GBA samples. Having said this, there was some variation in both the lipidomics^13^ and proteomics analyses that could be caused by technical issues but is more likely due to sample variation. The only information available about the samples is documented in ref. ^13^, and we cannot therefore exclude the possibility that some of the variation was due to factors beyond our control, such as co-pathologies, for example, in the IPD and PD-GBA samples. This kind of concern is inherent in the use of tissues samples obtained post-mortem.

As mentioned above, a number of proteomics studies have chosen specific brain regions (often the SN, in which significant neuronal loss occurs) to attempt to determine pathophysiological pathways in PD, and to delineate the biochemical pathways that link PD with *GBA1* mutations. In the case of PD^14,15^, a number of pathways were identified, including mitochondrial dysfunction (the electron transport chain and oxidative stress), indicating a critical role for mitochondrial (dys)function in PD. Other proteomics studies demonstrated the upregulation of extracellular matrix structural components and hemoglobin chains in PD patients^33^ (in Brodmann area 9 of the cortex), and determined a unique signature of proteins in PD patients related to the immune response^34^ (in the anterior CG). No proteomics studies to date have directly compared IPD with PD-GBA brain samples although one study examined PD-GBA samples^35^ alone using tissue from the anterior CG, and demonstrated reduced chaperone-mediated autophagy and elevation of lysosomal pathways.

In our current study, we analyzed 5 different brain areas, and saw significant differences in protein expression between brain areas in IPD and in PD-GBA. Whilst this is not unexpected, based on the different functions of different brain areas, it does nevertheless highlight the need to take brain areas other than the dopaminergic nigrostriatal into account when attempting to delineate PD-GBA pathology. This is particularly true for the brain areas associated with NMSs, not least because NMSs are considered more severe in PD-GBA than in IPD.

**Fig. 9:**
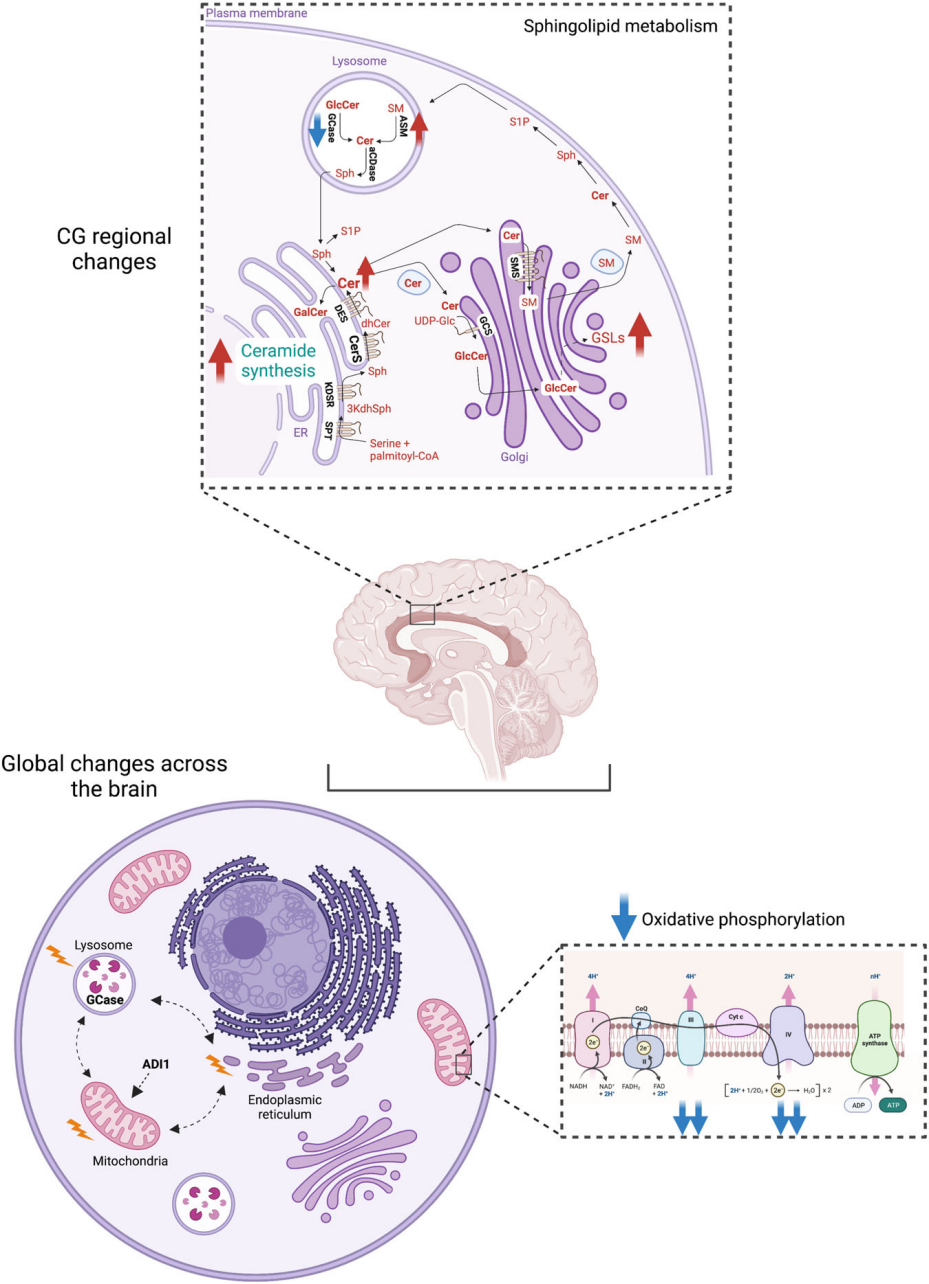
Summary of findings from the study. See text for further details. Created using BioRender.com.

Levels of only two proteins were altered in all 5 brain regions in either IPD or PD-GBA, namely GCase, which was lower in PD-GBA samples than in IPD, and ADl1, which was decreased in IPD but found at the same levels as controls in PD-GBA (Fig. 9). No data is available directly linking ADI1 to PD, although anecdotal evidence links ADI1 to a number of other diseases such as mitochondrial myopathy, encephalopathy, lactic acidosis, Alzheimer’s disease and stroke-like episodes. ADI1 is an iron-containing, acireductone dioxygenase involved in methionine metabolism which regulates a membrane-type 1 metalloproteinase (MMP14); recent studies showed an inverse correlation between levels of ADI1 and the development of pancreatic tumors^20^. ADI1 may also play a role in autophagy as ADI1 abrogates autophagy triggered by over-expression of MMP14^36^. MMP14 also regulates nerve regeneration^37^, and the interaction with ADI1 suggests that ADI1 may also indirectly play a role in axonal degeneration. Whether these pathways are related to PD is unclear, but the differences in ADI1 levels between IPD and PD-GBA may suggest that this protein plays an important regulatory role.

Two other potentially important findings can be derived from this dataset. The first involves the CG, in which a number of components of the sphingolipid biosynthetic pathway were altered (Fig. 9); the CG appears to be overactivated in PD by way of attempting to inhibit the abnormal and involuntary movements displayed in PD^26^. In general, these changes were more pronounced in PD-GBA than in IPD. Noticeably, levels of ceramide synthesis via two of the six CerSs isoforms^38^ were elevated (Table 1), which is in-line with changes in ceramide levels from our lipidomics analyses^13^ (Fig. 7). However, having said this, and despite the obvious attraction of changes in the ceramide synthesis pathway in a human disease genetically associated with mutations in an enzyme related to sphingolipid degradation (GCase), caution must be applied to these findings inasmuch as these effects were only seen in one brain area, and little is known about the sphingolipid pathway in human CG. Since the different CerSs are distributed in specific cell types in the brain^39^, care must be taken not to misattribute changes in CerS levels to a specific mechanistic pathway when the changes might simply be reflecting loss of specific brain cell types. Thus, while our data (including the lipidomics data) are suggestive of a role of ceramide biosynthesis in IPD/PD-GBA in the CG, additional supportive data should be obtained.

The other finding (Fig. 9) is that while changes in lysosomal proteins are entirely consistent with the notion that the lysosome plays a critical mechanistic role in PD, and in PD associated with mutations in lysosomal genes, levels of proteins in other cellular compartments are clearly altered in IPD and PD-GBA, including the endoplasmic reticulum, the plasma membrane and the mitochondria, in all five brain areas. Presumably these changes are secondary to the underlying cause of PD pathology, but since brain samples are only available post-mortem from human brain tissues, it is not possible to determine a time-course of the appearance of secondary versus primary pathological pathways. Even though a role of the mitochondria in (secondary) PD pathology has been suggested previously, our study now demonstrates of a role of this pathway, and in particular of the mitochondrial respiratory chain in human PD-GBA brain in a large-scale analysis, and suggests that this pathway may be more affected by directly comparing PD-GBA to IPD. Since PD-GBA is a more severe form of PD, this might not seem surprising, but it does nevertheless raise the issue of where altered mitochondrial function falls within the gamete of cellular modifications in biochemical pathways affected in PD-GBA.

In summary, this study not only presents data on putative pathological pathways in PD-GBA but also provides a resource documenting levels of ~ 5000 proteins in IPD and in PD-GBA in 5 different brain areas.

## Methods

### Human brain samples

Human brain samples were obtained as frozen tissue from the Queens Square Brain Bank of London, and included 21 IPD patients without a *GBA* mutation, 21 PD-GBA patients and 21 controls, from five different brain regions (OCC, MTG, CG, STR and SN). The CG region contained only 52 samples (15 controls, 18 IPD and 19 PD-GBA), the STR region contained 61 samples (19 controls, 21 IPD and 21 PD-GBA) and the SN region contained 60 samples (20 controls, 20 IPD and 20 PD-GBA), for a total of 299 samples. The groups in this study were age- and sex-matched. *GBA1* was sequenced by the Queens Square Brain Bank of London and *GBA* mutations for each of the PD-GBA patients has been documented^13^. The study was approved by the National Research Ethics Service (NRES) committee, London (127366). All participants gave written informed consent.

### Quantification of proteins by non-targeted and targeted proteomics

#### Sample preparation

All chemicals and reagents were from Sigma unless otherwise noted. Brain tissue samples were lysed with 5% sodium dodecyl sulfate (SDS) in 50 mM Tris-HCl. Lysates were incubated at 96 °C for 5 min, followed by six cycles of 30 sec of sonication (Bioruptor Pico, Diagenode, USA). Protein was determined using the BCA assay (Thermo Scientific, USA) and 100 μg protein was reduced with 5 mM dithiothreitol and alkylated with 10 mM iodoacetamide in the dark. Each sample was loaded onto S-Trap mini plates (Protifi, USA) according to manufacturer’s instructions. In brief, after loading, samples were washed with 90:10 methanol:50 mM ammonium bicarbonate. Samples were then digested with trypsin (1:50 trypsin/protein) for 1.5 h at 47 °C. The digested peptides were eluted using 50 mM ammonium bicarbonate; trypsin was added to this fraction and incubated overnight at 37 °C. Two more elutions were made using 0.2% formic acid and 0.2% formic acid in 50% acetonitrile. The three elutions were pooled together and vacuum-centrifuged to dry. Samples were kept at −80 °C until analysis.

#### Liquid chromatography

ULC/MS grade solvents were used for all chromatographic steps. Each sample was loaded using split-less nano-Ultra Performance Liquid Chromatography (nanoUPLC) (10 kpsi nanoAcquity; Waters, Milford, MA, USA). The mobile phase was A) H2O + 0.1% formic acid and B) acetonitrile + 0.1% formic acid. Desalting of the samples was performed online using a reversed-phase Symmetry C18 trapping column (180 µm internal diameter, 20 mm length, 5 µm particle size; Waters). Peptides were separated using a T3 HSS nano-column (75 µm internal diameter, 250 mm length, 1.8 µm particle size; Waters) at 0.35 µL/min. Peptides were eluted from the column into the mass spectrometer using the following gradient: 4 to 25% B for 150 min; 25 to 90% B for 5 min; maintained at 90% for 5 min and then back to initial conditions.

#### Mass spectrometry

The nanoUPLC was coupled online through a nanoESI emitter (10 μm tip; New Objective; Woburn, MA, USA) to a quadrupole orbitrap mass spectrometer (Q Exactive HFX, Thermo Scientific) using a FlexIon nanospray apparatus (Proxeon). Data was acquired in data-dependent acquisition (DDA) mode using the Top20 method. MS1 resolution was set to 120,000 (at 400 m/z), mass range of 375–1650 m/z, AGC of 3e6 and maximum injection time was set to 60 msec. MS2 resolution was set to 15,000, quadrupole isolation 1.7 m/z, AGC of 1e5, dynamic exclusion of 40 sec and maximum injection time of 60 msec. A preferential inclusion list was specified for higher priority of MS/MS triggering. The list of peptides is provided as Supplementary Data 1.

#### Data processing

Raw data was processed with MaxQuant version 1.6.0.16^40^. The data was searched with the Andromeda search engine against the Uniprot human proteome database (November 2018 version) appended with common laboratory protein contaminants and the following modifications: Carbamidomethylation of C as a fixed modification and oxidation of M, deamidation of N or Q and N-terminal acetylation as variables. Protein quantification was based on unique peptides with the minimal peptide ratio count set to 1 and a match between runs was enabled. The rest of the parameters were kept as default. LFQ intensities values from the protein groups file were used for analysis. Only proteins that were validated for at least 2/3 samples within each group were considered. Missing values were replaced with a bounded minimum value calculated as 3 standard deviations (SD) below the average intensity. For differential expression analysis, log2 intensities were analyzed using the ‘limma‘ package in R^41^, fitting the data into a linear model (lmFit function) and estimating the empirical Bayes statistics (eBayes function, robust=T). Differentially expressed proteins were determined by *p* ≤ 0.05 and fold-change ≥ 1.5.

#### Targeted proteomics

Targeted analysis was conducted on the same samples, which were spiked with heavy labeled synthetic peptides (see list of peptides and final concentrations in Supplementary Data 2). The same instrumentation and LC method were used as for data-dependent acquisition (DDA) runs but with a shorter gradient of 4 to 35% B for 105 min. Data was acquired in parallel reaction monitoring (PRM) mode, where MS2 resolution was set to 15,000, AGC target 2e5 and the maximum injection time set to 200 msec. Data analysis was performed using Skyline v20.1.0.76. The extracted MS2 intensities for each peptide were normalized to the total signal. Log2 transformation and a Student’s t-test were used to calculate the *p* value and fold-change between the 3 different groups. The average of all peptides per protein was used for the sample calculations. The heavy labeled counterpart peptide was used to verify the identification of the endogenous forms.

### Statistics

Both proteomics studies were analyzed using R version 3.6.1. See raw data for the analysis in Supplementary Data 3 and Supplementary Data 4. Principal Component Analysis (PCA) was used to analyze the influence of various factors on the data (age, gender and *GBA1* mutation). Samples were clustered to evaluate clustering into groups. Boxplots and Volcano plot were created using the ‘ggplot2’ package. Pearson Correlation matrices were created using the ‘GGally’ package. Venn diagrams were created in https://bioinformatics.psb.ugent.be/webtools/Venn/. For pathway analysis, data were analyzed using Ingenuity pathway analysis (IPA) (QIAGEN Inc., https://www.qiagenbioinformatics.com/products/ingenuitypathway-analysis). PERMANOVA (Adonis) with Euclidean distances on the subunits of each complex was used to examine mitochondrial complexes. The pairwise comparisons between the sample groups (i.e. control, IPD and PD-GBA) were performed using pairwise.perm.manova (RVAideMemoire). The statistical significance of proteomics data was evaluated as described above, and all other analyses were evaluated using ANOVA followed by post-hoc pairwise comparisons using the Tukey’s honest significant difference test (Tukey HSD).

### Analysis of β-sheet oligomers of human α-synuclein

Brain tissue was homogenized in 500 μl radioimmunoprecipitation assay (RIPA) lysis buffer (EMD Millipore, Massachusetts, USA) containing 1:100 vol:vol protease inhibitor cocktail (MilliporeSigma, Massachusetts, USA) using a gentle MACS Octo Dissociator (Miltenyi Biotec, Bergisch Gladbach, Germany). Protein concentration was estimated using the QPRO-BCA reagent (Cyanagen SRL, Bologna, Italy) and β-sheet oligomers of α-synuclein were measured by the human alpha-Synuclein PATHO ELISA (Roboscreen GmbH, Leipzig, Germany^42^). Quantification was performed using the Infinite M Plex plate reader (Tecan, Männedorf, Switzerland).

### GCase activity

GCase activity was determined as previously described^43^. Brain tissue was homogenized in 200 μl McIlvaine’s buffer (0.1 M citric acid, pH 4.2, 0.2 M Na_2_HPO_4_)^44^ and 1:100 vol:vol protease inhibitor cocktail (MilliporeSigma, Massachusetts, USA). Protein concentration was estimated using the QPRO-BCA reagent (Cyanagen SRL, Bologna, Italy). Brain homogenates were incubated at 37 °C with 20 μM C6-NBD-GlcCer^45^ (Matreya LLC, Pleasant Gap, PA, USA) in a final volume of 20 μl McIlvaine’s buffer for 1 h. Reactions were terminated by addition of three volumes of chloroform:methanol (1:2, vol:vol). Lipids were extracted^46^ and separated by thin layer chromatography using chloroform:methanol:9.8 mM CaCl_2_ (60:35:8, vol:vol:vol) as the developing solvent. NBD-lipids were quantified using Amersham Typhoon 5 biomolecular fluorescence imager and quantified by ImageQuantTL (GE Healthcare, Chalfont St Giles, UK).

### CerS assay

CerS activity was assayed as described^47,48^. Brain samples were homogenized in 20 mM Hepes–KOH, pH 7.2, 25 mM KCl, 250 mM sucrose, and 2 mM MgCl2 containing 1:100 vol:vol of a protease inhibitor cocktail. Protein concentration was estimated using the QPRO-BCA reagent. Brain homogenates were incubated with 15 μM NBD-Sph, 20 μM defatted-bovine serum albumin and 50 μM fatty acyl CoA in a 20 μl reaction volume at 37 °C, for either 30 min or 1 h, depending on the fatty acyl CoA. Reactions were terminated by the addition of chloroform/methanol (1:2, v/v) and lipids extracted. Lipids were dried under N_2_ and resuspended in chloroform/methanol (9:1, v/v) and applied to thin layer chromatography plates, which were subsequently developed using chloroform/methanol/2 M NH4OH (40:10:1, v/v/v). NBD-labeled lipids were visualized using an Amersham Typhoon 5 biomolecular fluorescence imager and quantified by ImageQuantTL.

### Supplementary information

Supplementary material

Supplementary Data 1

Supplementary Data 2

Supplementary Data 3

Supplementary Data 4

nr-reporting-summary

## Data Availability

The authors declare that all data supporting the findings of this study are available within the paper and the supplementary information. Data are available via ProteomeXchange with identifier PXD047134.
