# Supplementary material for: Large-scale proteomics analysis of five brain regions from Parkinson’s disease patients with a GBA1 mutation
Source: NPJ Parkinsons Dis. 2024 Feb 8;10:33. doi: 10.1038/s41531-024-00645-x (PMC10853186; doi:10.1038/s41531-024-00645-x)
Supplement: Supplementary file 1 — Supplementary material [file 41531_2024_645_MOESM1_ESM.pdf]

Large-scale proteomics analysis of five brain regions from Parkinson's disease patients with a *GBA1* mutation

Shani Blumenreich[‡], Tamar Nehushtan[‡], Meital Kupervaser[Ψ], Tali Shalit[Ψ], Alexandra Gabashvili[Ψ], Tammar Joseph[‡], Ivan Milenkovic[‡,@], John Hardy[#] and Anthony H. Futerman[‡,||,*]

Supplemental Information

Supplementary Figure 1. *Comparison between targeted and non-targeted proteomics.* Pearson correlation matrices of 14 proteins out of 17 proteins measured by non-targeted and targeted proteomics from the CG, are shown by way of example. ADI1 was removed from this analysis as it was identified with only one peptide in the targeted analysis, TH was not identified in the non-targeted analysis in this region and LPCAT2 was removed due to a technical issue. Both axes are in arbitrary units. Control, *red*; IPD, *green*; PD-GBA, *blue*. The relatively high R and *p* values (<0.004) indicate validation of the non-targeted proteomics by the targeted proteomics.

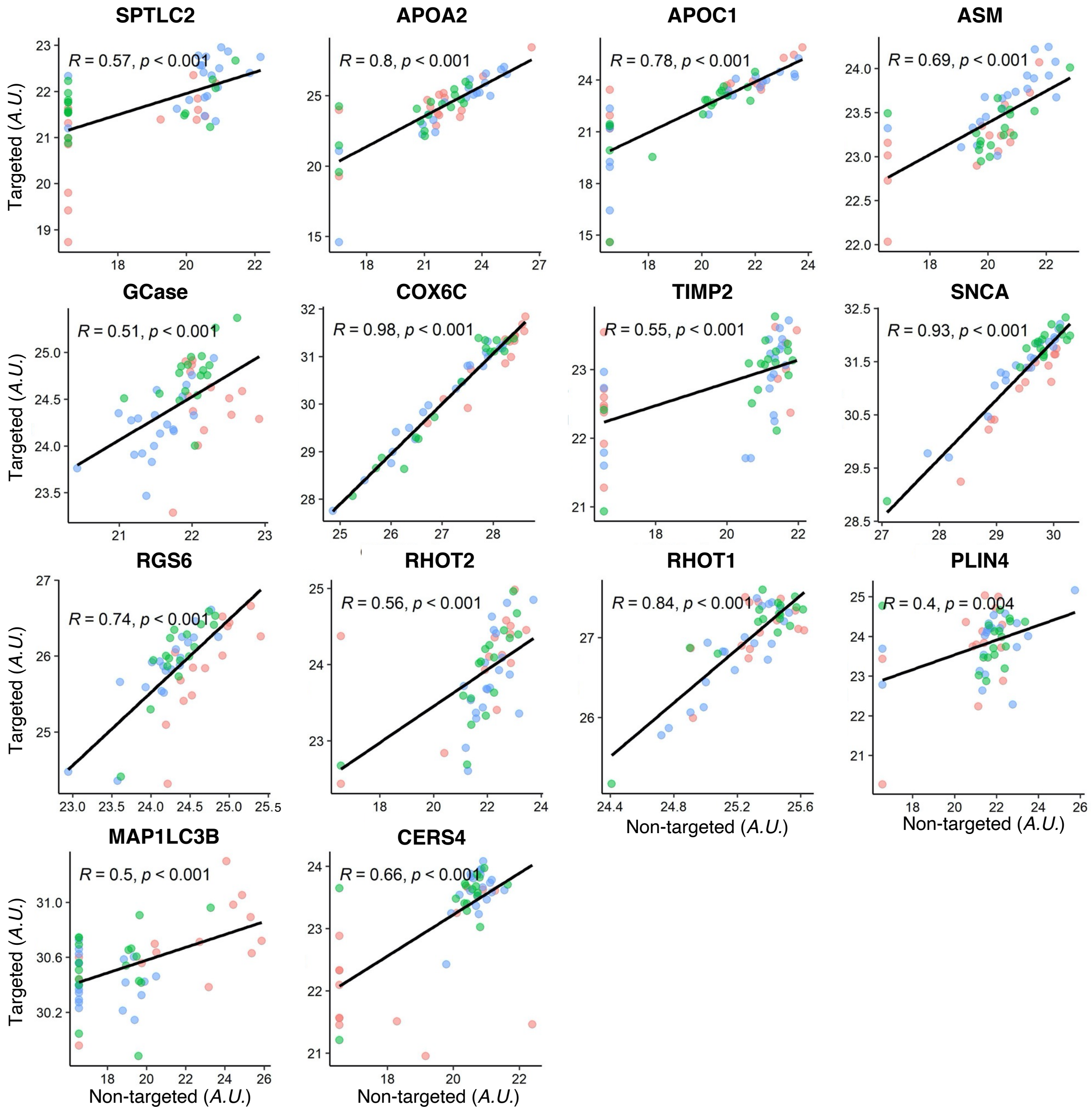

Supplementary Figure 2. *Evaluation of the quality of the proteomics dataset.* Non-targeted, mass spectrometry based quantitative discovery proteomics was performed on five human brain regions, namely the OCC, MTG, CG, STR and SN of 21 control, 21 IPD and 21 PD-GBA samples. PCA plots showing sample clustering according to gender, age and *GBA1* mutation. Controls, *circles*; IPD, *triangles*; PD-GBA, *squares*. Color-coding is documented and indicates age, gender or *GBA1* mutation. The axes represent sample variance. No correlation was observed for any of these factors.

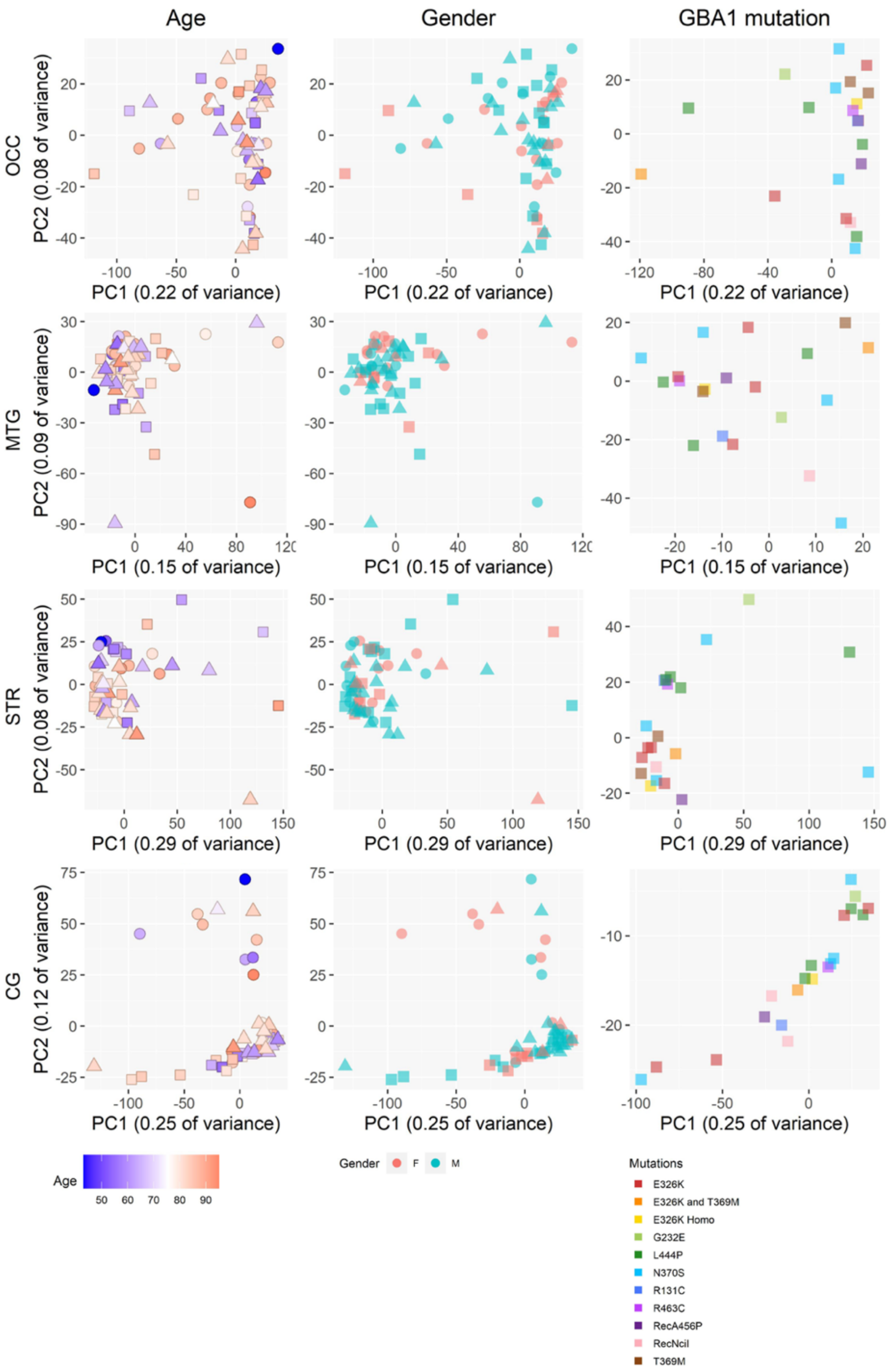

Supplementary Figure 3. *Additional evaluation of the quality of the proteomics dataset by principal component analysis.* PCA plots showing sample clustering according to gender, age and *GBA1* mutation of PC3 and PC4 explained variance. Controls, *circles*; IPD, *triangles*; PD-GBA, *squares*. Color-coding is documented and indicates age, gender or *GBA1* mutation. The axes represent sample variance. No correlation was observed for any of these factors.

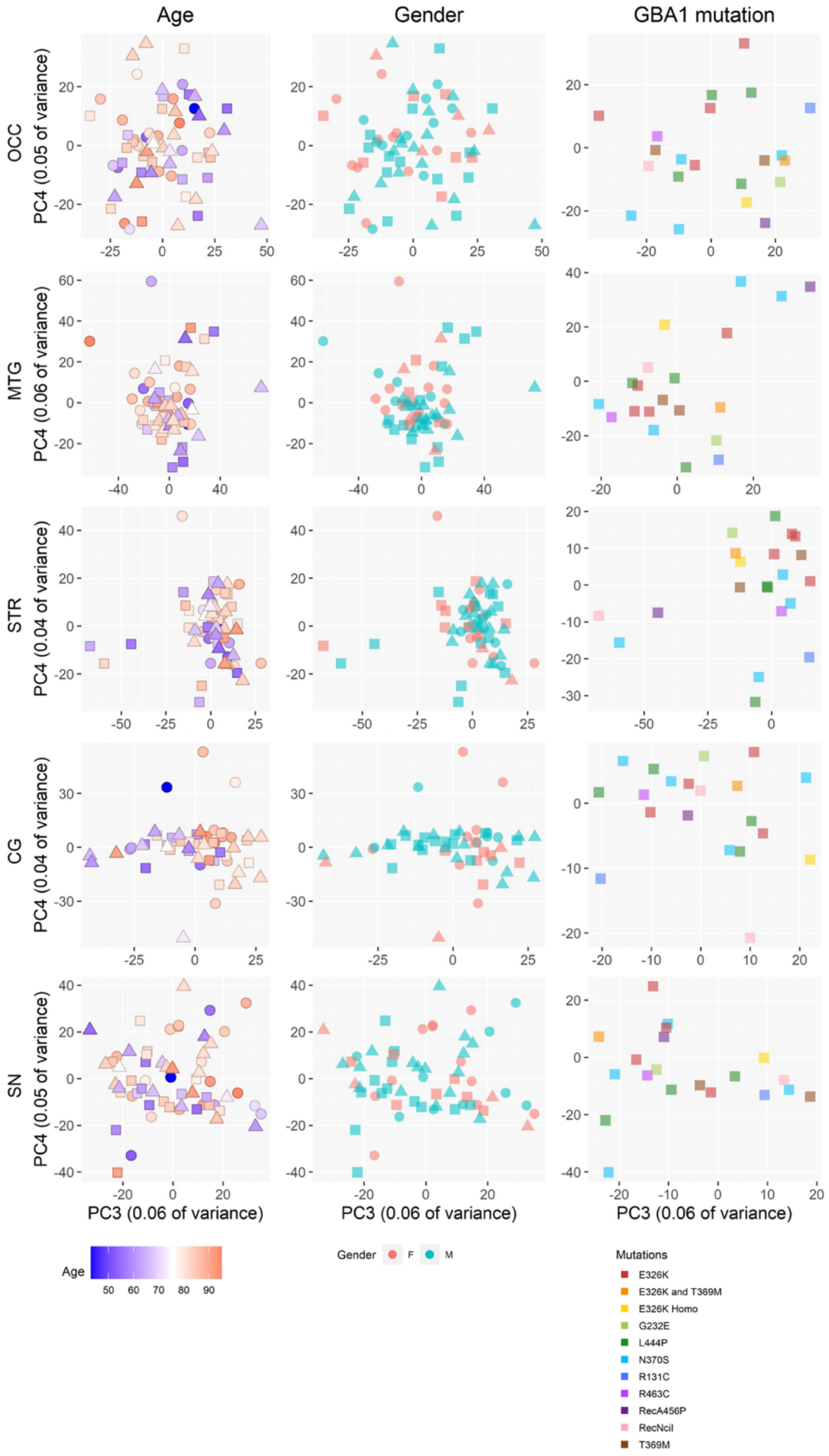

Supplementary Figure 4. *Evaluation of sample quality.* Non-targeted, mass spectrometry based quantitative discovery proteomics was performed on five human brain regions, namely the OCC, MTG, CG, STR and SN of 21 control, 21 IPD and 21 PD-GBA samples. The number of missing values per sample across all brain regions is shown. The horizontal line represents the average of missing values across all samples. Due to the high number of missing values, sample PG11 from the MTG and samples C13 and C15 from the CG were defined as outliers and excluded from further analysis. Control, *red*; IPD, *blue*; PD-GBA, *green*. Y axis represents the number of missing values, x axis represents individual samples.

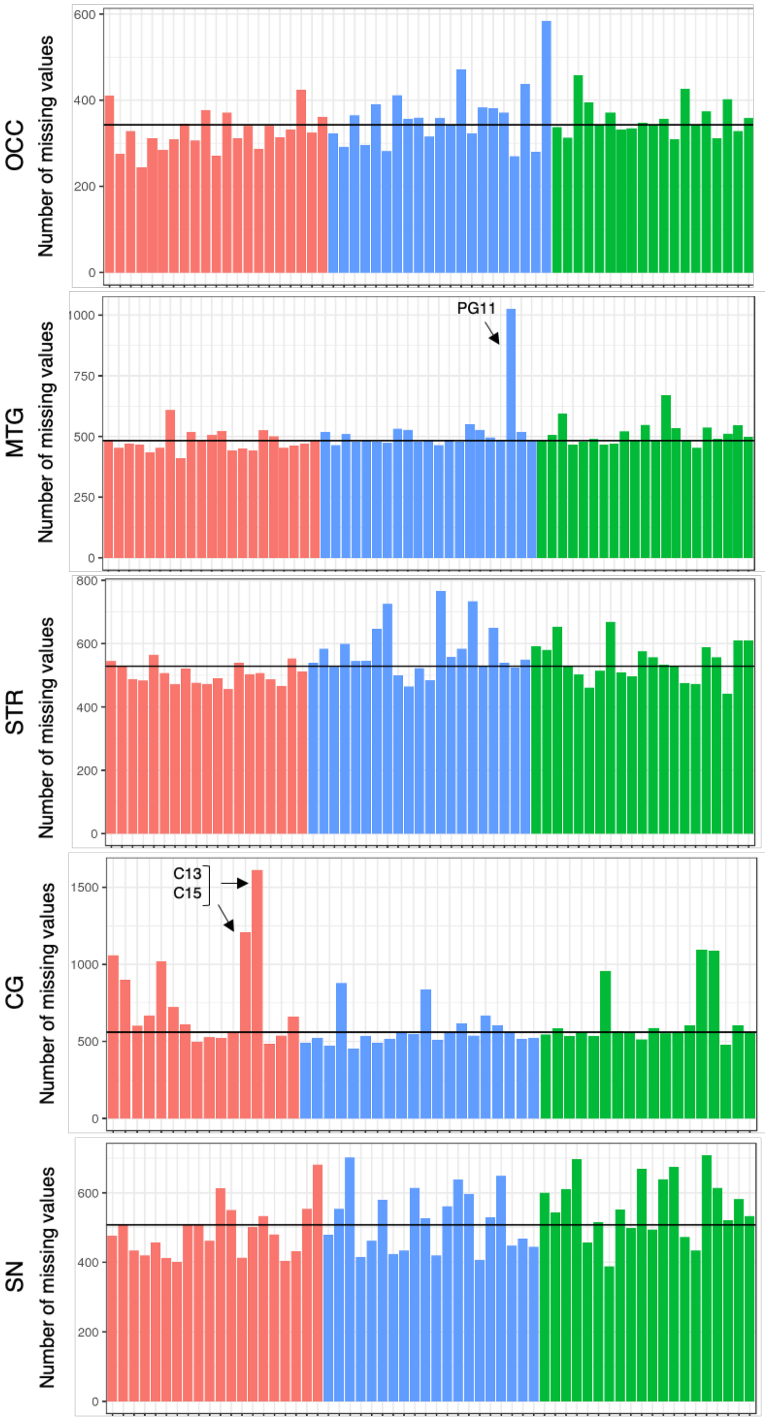

Supplementary Figure 5. *Sample clustering based on 3938 proteins detected in all brain regions.* Heatmap showing sample clustering for the SN as an example (this is an extension of data shown in Fig. 2B). The heatmap displays z-scores for all 3938 proteins. Z-scores above zero are shown in *red* and z-scores below zero are shown in *blue*. No clear clustering into sample groups (i.e. control; C, IPD; PD or PD-GBA; PG) was observed.

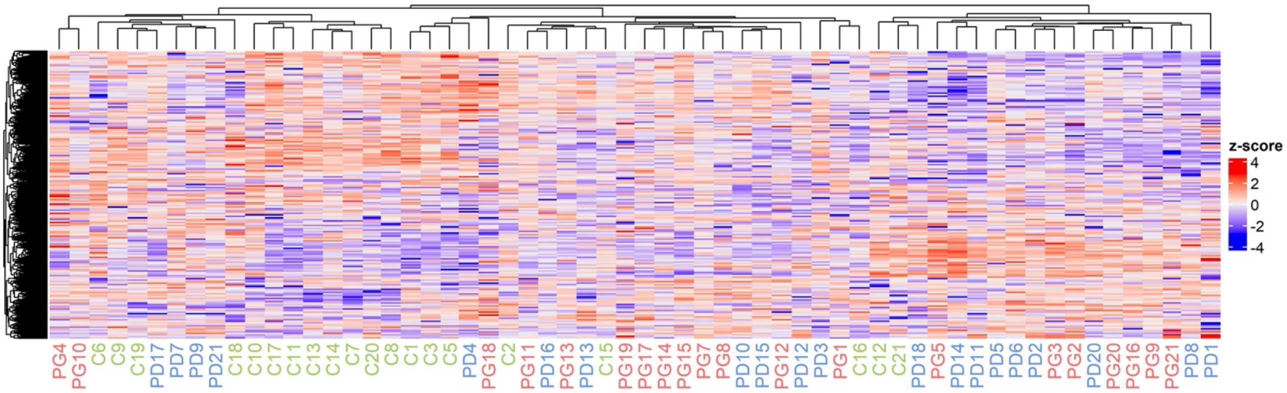

Supplementary Figure 6. *Validation of changes in TH levels in targeted proteomics.* Pearson correlation matrices of TH in the non-targeted and targeted proteomics. Correlation coefficient values comparing the the non-targeted (*x axis*) *versus* the targeted (*y axis*) proteomics data. Both axes are in arbitrary units. Control, *red*; IPD, *green*; PD-GBA, *blue*.

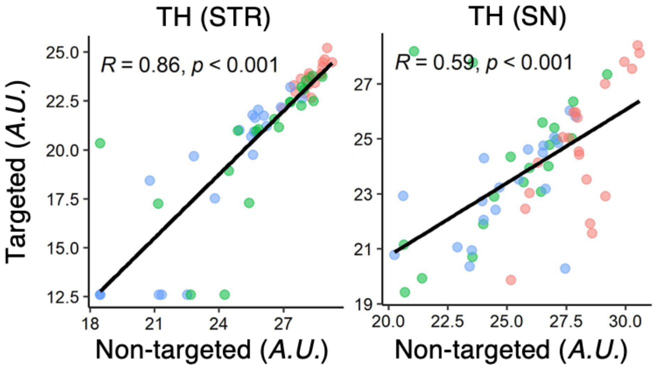

Supplementary Figure 7. *GCase validation in targeted proteomics.* Boxplots displaying targeted proteomics of GCase levels in all brain regions measured in the analysis. The box represents lower quartile, median and upper quartile (*black*). The whiskers represent the minimum and maximum values, up to 1.5 times the interquartile range from the bottom or the top of the box to the furthest data point within that distance, thus excluding outliers. The mean is shown in *red*. The y axis is in arbitrary units. *$p \leq 0.05$; **$p \leq 0.01$; ***$p \leq 0.001$, calculated using empirical Bayes moderation.

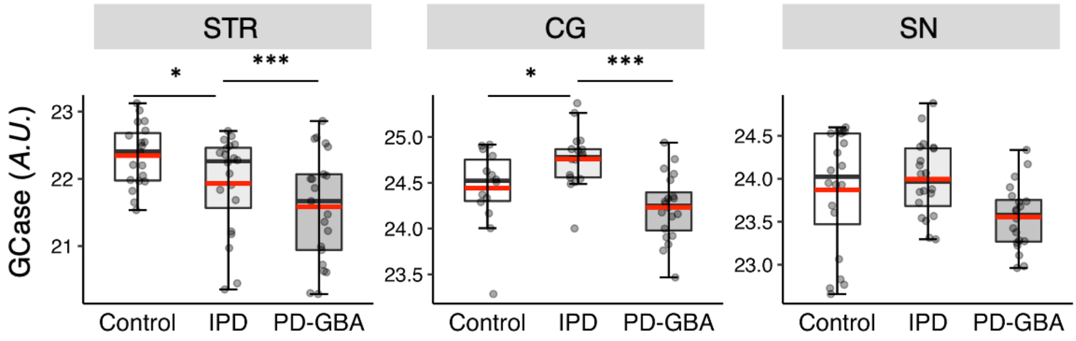

Supplementary Figure 8. *Lack of correlation between GCase levels and age.* Scatter plot of GCase levels from non-targeted proteomics and age for the control group. R^2 value is indicated. Y axis is in arbitrary units.

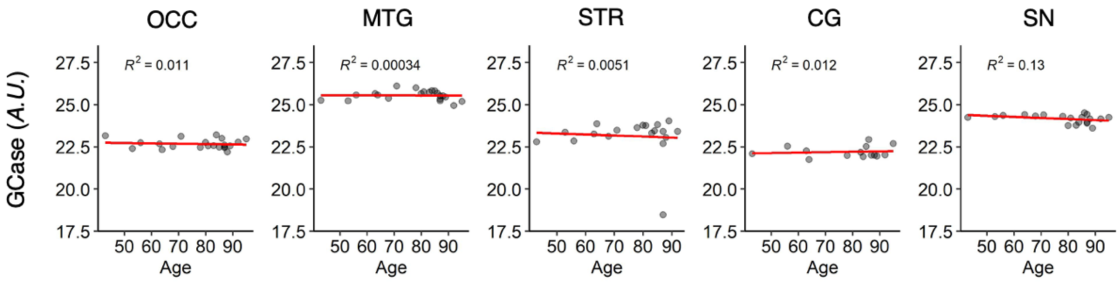

Supplementary Figure 9. Validation of *COX6C levels by targeted proteomics.* Pearson correlation matrices of COX6C levels in non-targeted and targeted proteomics from the CG (*left panel*) and SN (*right panel*). Correlation coefficient values are indicated. Axes represent the non-targeted (*x axis*) *versus* the targeted (*y axis*) proteomics results. Both axes are in arbitrary units. Control, *red*; IPD, *green*; PD-GBA, *blue*.

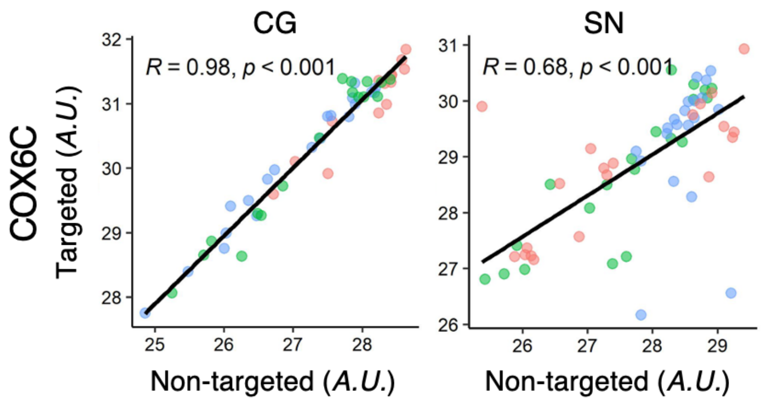

Supplementary Figure 10. *Validation of ceramide synthesis related proteins in targeted proteomics*. *A,* Boxplots indicating *levels of* SPTLC2 by targeted proteomics. *B,* Boxplot of targeted proteomics for CerS4. For all boxplots, the box represents lower quartile, median and upper quartile (*black*). The whiskers represent the minimum and maximum values, up to 1.5 times the interquartile range from the bottom or the top of the box to the furthest data point within that distance, thus excluding outliers. The mean is shown in *red*. **p*≤0.05, ***p*≤0.01, ****p*≤0.001, calculated using empirical Bayes moderation.

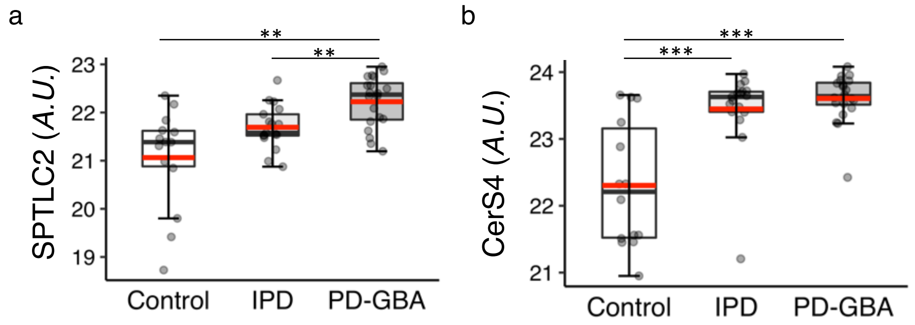

Supplementary Figure 11. *ASM levels in the CG by targeted proteomics.* Boxplots of ASM levels in the CG by targeted proteomics. The box represents lower quartile, median and upper quartile (*black*). The whiskers represent the minimum and maximum values, up to 1.5 times the interquartile range from the bottom or the top of the box to the furthest data point within that distance, thus excluding outliers. The mean is shown in *red*. Y axis is in arbitrary units. *$p \leq 0.05$, **$p \leq 0.01$, ***$p \leq 0.001$, calculated using empirical Bayes moderation.

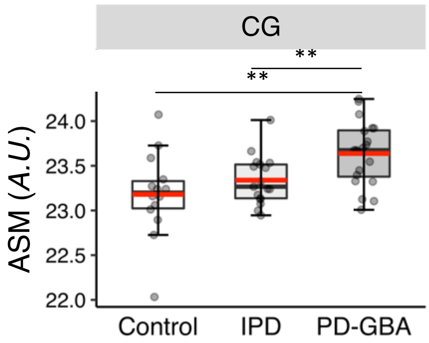

Supplementary Table 1. *Levels of ceramide synthesis-related proteins*. Ceramide synthesis-related proteins in four brain regions (see Table 1 for the CG). Ratios of protein levels from the non-targeted proteomics results are shown for IPD *versus* control, PD-GBA *versus* control and PD-GBA *versus* IPD. *$p \leq 0.05$; **$p \leq 0.01$, calculated using empirical Bayes moderation.

		OCC			MTG			STR			SN		
Gene	Protein	Ratio (IPD vs control)	Ratio (PD-GBA vs control)	Ratio (PD-GBA vs IPD)	Ratio (IPD vs control)	Ratio (PD-GBA vs control)	Ratio (PD-GBA vs IPD)	Ratio (IPD vs control)	Ratio (PD-GBA vs control)	Ratio (PD-GBA vs IPD)	Ratio (IPD vs control)	Ratio (PD-GBA vs control)	Ratio (PD-GBA vs IPD)
SPTLC1	Serine palmitoyltransferase, long chain base subunit 1				0.66	0.74	1.11	1.12	0.89	0.80	0.42	0.46	1.11
SPTLC2	Serine palmitoyltransferase, long chain base subunit 2												
KDSR	3-ketodihydrosphingosine reductase	0.98	1.03	1.05	0.95	0.94	1.00	1.06	1.06	1.00	1.06	0.97	0.92*
CERS1	Ceramide synthase 1	0.94	1.00	1.06	0.98	0.89	0.91	1.19	1.09	0.92	1.04	0.95	0.92
CERS2	Ceramide synthase 2	0.89	0.91	1.03	0.84	0.52	0.62	0.94	0.92	0.98	1.30**	1.25*	0.96
CERS4	Ceramide synthase 4				1.42	1.13	0.80	0.76	1.21	1.60*			
CERS6	Ceramide synthase 6				1.00	1.18	1.18	0.97	0.73	0.75	0.59	0.82	1.39
DEGS1	Sphingolipid delta(4)-desaturase DES1	0.99	0.97	0.99	1.10	0.97	0.88	1.02	1.04	1.02	0.72	0.80	1.11

Supplementary Table 2. *Alterations in LSD-related proteins in both IPD and PD-GBA brain samples. This data is presented in Fig. 8 as a heat-map.* Ratios are indicated. Blanks are non-available samples. *$p \leq 0.05$, **$p \leq 0.01$, ***$p \leq 0.001$, calculated using empirical Bayes moderation.

		OCC			MTG			STR			CG			SN		
Gene	Protein	Ratio (IPD vs control)	Ratio (PD-GBA vs control)	Ratio (PD-GBA vs IPD)	Ratio (IPD vs control)	Ratio (PD-GBA vs control)	Ratio (PD-GBA vs IPD)	Ratio (IPD vs control)	Ratio (PD-GBA vs control)	Ratio (PD-GBA vs IPD)	Ratio (IPD vs control)	Ratio (PD-GBA vs control)	Ratio (PD-GBA vs IPD)	Ratio (IPD vs control)	Ratio (PD-GBA vs control)	Ratio (PD-GBA vs IPD)
ARSB	ARSB	0.94	0.93	0.99	0.89	0.93	1.05	1.05	1.27*	1.22	0.94	0.84	0.90	0.57	0.41	0.72
ASAH1	ASAH1	0.97	0.98	1.01	1.01	0.95	0.94	1.12	1.17*	1.05	1.07	1.24	1.16	0.94	0.90	0.96
CTSA	PPGB	0.78	0.72*	0.93	0.94	0.85**	0.91	1.01	1.05	1.05	1.84*	2.32**	1.26	0.94	0.64	0.68
CTSD	CATD	0.94	0.89**	0.94	0.91	0.85**	0.93	1.04	1.03	0.99	0.95	0.97	1.02	0.76***	0.72***	0.95
DNAJC5	DNJC5	1.05	1.01	0.97	1.12	1.12	1.00	0.93	0.90	0.97	1.63**	1.41*	0.86	0.92	1.11	1.21
GAA	LYAG	*0.86	0.90	1.05	0.89	0.87*	0.97	1.04	1.04	0.99	0.75**	0.75**	1.00	0.90	0.87*	0.96
GALC	GALC	1.39	1.49	1.07	0.76	0.82	1.09	1.12	1.33	1.18	0.95	3.46*	3.65**	1.24	1.22	0.98
GBA1	GCase	0.96	0.40***	0.41***	0.83	0.51***	0.61**	1.05	0.83	0.79	0.86	0.63***	0.73***	0.89	0.53***	0.59**
GLA	AGAL				0.79	0.53	0.67							0.43*	0.45*	1.05
GLB1	BGAL	1.01	1.00	1.00	0.82**	0.80**	0.98	1.01	0.94	0.93	0.60*	0.81	1.34	0.70***	0.61***	0.87
GM2A	SAP3	0.92	0.97	1.06	1.06	1.00	0.95	1.01	1.15	1.15	1.32	1.89*	1.43	1.17*	1.07	0.91
GNS	GNS	0.93	0.88	0.95	0.98	0.89	0.91	1.04	1.17	1.12	2.39	3.39**	1.42	1.02	0.98	0.96
HEXA	HEXA	*0.80	0.93	1.16	0.83*	0.90	1.08	0.84	0.99	1.18	0.78*	0.88	1.12	0.71***	0.73***	1.04
IDS	IDS				1.05	0.73	0.69*				0.76	0.48**	0.62*	0.39***	0.40***	1.01
KCTD7	KCTD7	0.78	0.62*	0.79				0.80	0.76	0.95				0.90	0.74	0.82
LAMP2	LAMP2	0.93	0.95	1.03	1.00	0.92	0.92	0.98	1.22	1.25*	1.06	1.47*	1.39*	1.10	0.96	0.87
LIPA	LICH	0.98	0.72	0.74	0.81	0.59**	0.72	0.98	1.14	1.16	0.81	1.23	1.51*	1.20*	1.13	0.94
NAGA	NAGAB				0.85	1.05	1.23				0.47	1.28	2.71*	0.52	0.47	0.90
NPC1	NPC1	0.76*	0.89	1.17	0.70	0.75	1.08	0.85	0.92	1.08	0.93	1.41*	1.51**	1.05	0.98	0.93
NPC2	NPC2	0.91	0.94	1.03	0.99	0.98	0.99	0.99	1.00	1.01	1.09	1.42*	1.30	1.07	0.97	0.90
PSAP	SAP	0.92	0.98	1.06	1.03	1.00	0.97	0.94	1.12	1.19*	0.81	1.02	1.26	1.23*	1.35**	1.09
SCARB2	SCRB2	0.98	1.03	1.04	1.04	1.03	0.99	1.05	1.10	1.05	1.05	1.25**	1.19*	0.98	0.99	1.00
SMPD1	ASM	0.41**	0.74	1.78	0.84	0.68*	0.81	0.99	1.11	1.12	1.53	2.27*	1.48	1.07	1.03	0.96

Supplementary Table 3. *Number of identified proteins in cell compartments from all five brain regions.* See Table 2 for further details. The number of total proteins is given in Table 2.

Brain region	Mitochondria	Endoplasmic reticulum	Lysosome	Plasma membrane
OCC	458	200	211	1665
MTG	526	228	235	1937
STR	522	209	225	1839
CG	531	227	238	1923
SN	466	216	215	1661

Supplementary Data 1. See attached excel file of inclusion peptides for *GBA1* and *SNCA* for non-targeted proteomics.

Supplementary Data 2. See attached excel file of list of peptides and their concentration (3 peptides for each of the 20 chosen proteins) used for targeted proteomics.

Supplementary Data 3. See attached excel file for the raw data of the non-targeted proteomics after logarithmic transformation.

Supplementary Data 4. See attached excel file of the raw data of the targeted proteomics after logarithmic transformation. Cases which were identified with only one peptide: In the CG, ADI1, TFEB, and TH; in the STR, ADI1, ASM and MMP14; in the SN, ADI1, TFEB and TIMP2.
